# Interaction between intestinal mycobiota and microbiota shapes lung inflammation

**DOI:** 10.1002/imt2.241

**Published:** 2024-09-14

**Authors:** Youxia Wang, Fang He, Bingnan Liu, Xiaoyan Wu, Ziyi Han, Xuefei Wang, Yuexia Liao, Jielin Duan, Wenkai Ren

**Affiliations:** ^1^ State Key Laboratory of Swine and Poultry Breeding Industry, College of Animal Science South China Agricultural University Guangzhou China; ^2^ College of Veterinary Medicine Southwest University Chongqing China; ^3^ School of Basic Medical Sciences Capital Medical University Beijing China; ^4^ School of Nursing & School of Public Health Yangzhou University Yangzhou China; ^5^ Department of Allergy and Clinical Immunology, State Key Laboratory of Respiratory Disease, National Clinical Research Center for Respiratory Disease, Guangzhou Institute of Respiratory Health The First Affiliated Hospital of Guangzhou Medical University Guangzhou China

**Keywords:** intestinal microbiota, intestinal mycrobiota, lung inflammation, macrophages

## Abstract

Gut microbiota is an intricate microbial community containing bacteria, fungi, viruses, archaea, and protozoa, and each of them contributes to diverse aspects of host health. Nevertheless, the influence of interaction among gut microbiota on host health remains uncovered. Here, we showed that the interaction between intestinal fungi and bacteria shaped lung inflammation during infection. Specifically, antifungal drug‐induced dysbiosis of gut mycobiota enhanced lung inflammation during infection. Dysbiosis of gut mycobiota led to gut *Escherichia coli* (*E. coli*) overgrowth and translocation to the lung during infection, which induced lung accumulation of the CD45^+^F4/80^+^Ly6G^−^Ly6C^−^CD11b^+^CD11c^+^ macrophages. Clearance of macrophages or deletion of TLR4 (Toll‐like receptor 4, recognition of LPS) rather than Dectin‐1 (recognition of beta‐1,3/1,6 glucans on fungi) blocked the antifungal drug‐induced aggravation of lung inflammation during infection. These findings suggest that the interaction between intestinal mycobiota and commensal bacteria affects host health through the gut–lung axis, offering a potential therapeutic target for ameliorating lung inflammation during infection.

## Highlights

- Antifungal drug‐induced dysbiosis of gut mycobiota aggravates lung inflammation during infection.
- Dysbiosis of intestinal mycobiota results in gut *Escherichia coli* overgrowth and translocation to the lung during infection.
- *E. coli* induces lung accumulation of the CD45^+^F4/80^+^Ly6G^−^Ly6C^−^CD11b^+^CD11c^+^ macrophages.
- Clearance of macrophages or deletion of TLR4 blocks the antifungal drug‐induced aggravation of lung inflammation during infection.

## Graphical abstract

Antifungal drug‐induced dysbiosis of gut mycobiota results in aggravation of lung inflammation during infection. Specifically, dysbiosis of gut mycobiota results in gut *Escherichia coli* overgrowth and translocation to the lung during infection. *E. coli* induces lung accumulation of the CD45^+^F4/80^+^Ly6G^−^Ly6C^−^CD11b^+^CD11c^+^ macrophages and activates TLR4 signaling of the CD45^+^F4/80^+^Ly6G^−^Ly6C^−^CD11b^+^CD11c^+^ macrophages. This study provided evidence for the interaction among gut inhabitants shaping the host immunity and diseases.

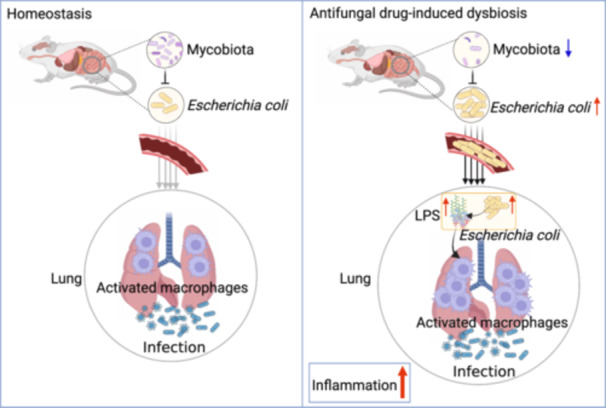

## INTRODUCTION

The intestine is a particularly intricate ecosystem comprising a diverse array of microorganisms, including bacteria, archaea, fungi, viruses, and protozoa [1]. Through millions of years of concomitant evolution and symbiosis with its host [2, 3], the gut microbiota contributes to diverse aspects of host health, including metabolism, homeostasis, and immune response [4]. Accumulating evidence suggests that gut microbiota dysbiosis impacts immune response in the lung through the gut–lung axis, and the underlying mechanisms may be intestinal barrier destruction and bacterial translocation [5, 6]. Research on gut microbiota dysbiosis mainly focuses on the disorder of intestinal bacteria, while the role of intestinal fungi, an essential component of gut microbiota, cannot be ignored [7]. Gut fungal dysbiosis also affects the immune response and pathological processes in the lung. For example, fungal dysbiosis increases lung group 2 innate lymphoid cells (ILC2) and enhances eosinophilic airway inflammation in mice [8]; and fungal imbalance is related to the course of COVID‐19 [9]. However, the underlying mechanisms by which gut fungal dysbiosis affects the immune response in the lung are yet to be known.

The combined effect of microbial interactions on host health has gained significant attention in recent years, with a particular focus on the interactions between fungi and bacteria. A strong functional connection exists between bacterial and fungal communities in the gut [10, 11], with fungi and bacteria influencing each other in a mutualistic or antagonistic manner to play important roles in host diseases [12, 13]. For instance, *Escherichia coli* promotes the virulence of *Candida albicans* (*C. albicans*), while *Bacteroidetes* and *Firmicutes* inhibit the colonization of *C. albicans* [14]. Furthermore, antibiotic‐triggered gut bacterial dysbiosis is associated with the expansion of *Candida* species [15]. Consistently, *Mucor circinelloides* and *C. albicans* have been found to increase the colonization of pathobiont bacteria while reducing the colonization of probiotic bacteria [16, 17]. Additionally, gut mycobiota has been shown to promote the growth of *Salmonella enterica* by producing siderophores, while inhibiting the growth of *E. coli* by producing antibacterial compounds [18, 19]. Notably, the interaction between gut mycobiota and commensal bacteria is closely linked to intestinal diseases, such as inflammatory bowel diseases (IBD) [20]. However, the influence of this interaction on extraintestinal diseases, such as lung inflammation, is still poorly understood.

Here, we aimed to elucidate that the interaction between intestinal fungi and bacteria shaped lung inflammation during infection through the gut–lung axis. This study provided evidence for the interaction among gut inhabitants shaping the host immunity and diseases.

## RESULTS

### Dysbiosis of intestinal mycobiota exacerbates lung inflammation during infection

**Figure 1:**
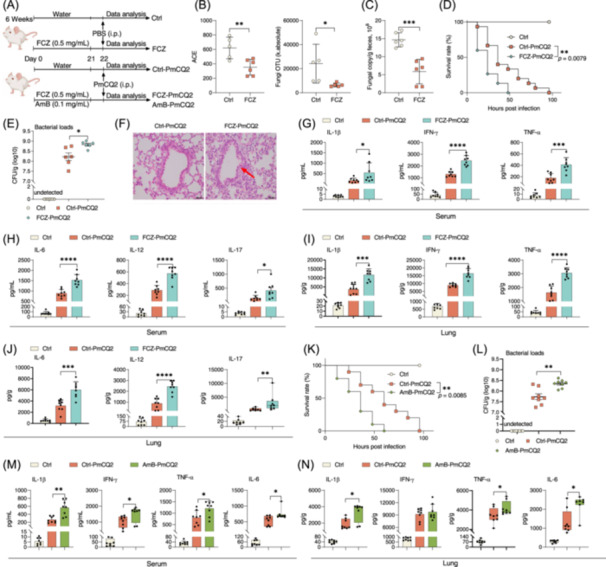
Antifungal drugs aggravate lung inflammation during infection. (A) Experimental design for fluconazole (FCZ) treatment, amphotericin‐B (AmB) treatment, and *Pasteurella multocida* type‐A (PmCQ2) infection. (B) Alpha‐diversity of intestinal mycobiota of mice. ACE, abundance‐based coverage estimator (*n* = 5 and 6). (C) Quantification of absolute abundance of the fungal populations (*n* = 6). (D) The survival rate of mice analyzed by Log‐rank test (*n* = 15). (E) Bacterial loads of mouse lung shown as mean ± SEM (*n* = 6). (F) The inflammation in the lung analyzed with hematoxylin–eosin (HE) staining (scale bars, 50 μm). (G–J) The levels of interleukin‐1β (IL‐1β), interferon‐γ (IFN‐γ), tumor necrosis factor‐α (TNF‐α), IL‐6, IL‐12, and IL‐17 in the serum and lung of mice (*n* = 8). The data of lung IL‐17 were analyzed by Mann–Whitney *U* test and shown as median with interquartile range (*M*(IQR)). (K) The survival rate of mice analyzed by Log‐rank test (*n* = 10). (L) Bacterial loads of mouse lung shown as mean ± SEM (*n* = 8). (M, N) The levels of IL‐1β, IFN‐γ, TNF‐α, and IL‐6 in the serum and lung of mice (*n* = 7 and 8). The data about serum IFN‐γ and IL‐6, and lung IL‐1β, TNF‐α as well as IL‐6 were analyzed by Mann–Whitney *U* test and shown as *M*(IQR). Data were analyzed by unpaired *t*‐test and represented as mean ± SD unless indicated. *p < 0.05, **p < 0.01, ***p < 0.001, and ****p < 0.0001.

To uncover the impact of intestinal mycobiota on immune response in extraintestinal organs, we first established an intestinal mycobiota dysbiosis model induced by fluconazole (an antifungal agent) (Figure 1A). Fluconazole effectively lowered the richness and absolute population of intestinal mycobiota (Figure 1B,C). Then, mice pretreated with fluconazole or not were infected by *Pasteurella multocida* type‐A (PmCQ2, a bacterium, that induces pneumonia) as previous methods [21, 22] (Figure 1A). Intriguingly, the overall survival rate of the mice pretreated with fluconazole was markedly reduced (Figure 1D), and the bacterial loads of PmCQ2 in the lung of fluconazole‐treated mice were significantly higher compared with the PmCQ2‐infected group (Figure 1E). Furthermore, we performed a histological assessment of lung inflammatory cell infiltration and observed an increase in immune cell infiltration in the lung tissues of fluconazole‐pretreated mice (Figure 1F). Likewise, pretreatment of fluconazole increased the levels of inflammatory cytokines, including interleukin‐1β (IL‐1β), interferon‐γ (IFN‐γ), tumor necrosis factor‐α (TNF‐α), IL‐6, IL‐12, and IL‐17, both in the serum and lung (Figure 1G−J). However, the mRNA expression of these cytokines, except for *Il10*, was not altered (Figure S1A–G), suggesting a posttranslational regulation for these cytokines in this context. These results suggest that fluconazole‐induced dysbiosis of intestinal mycobiota aggravates lung inflammation during PmCQ2 infection.

To investigate whether the observed mycobiota dysbiosis‐aggravated lung inflammation is specific for PmCQ2 infection, we employed a separate bacterial pathogen, *Streptococcus pneumoniae* (*S. pneumonia*), a prevalent cause of community‐acquired pneumonia [23]. While the results were not statistically significant, fluconazole pretreatment showed a similar trend as in the model of PmCQ2 infection, with a reduction in survival rate and an increase in lung bacteria loads (Figure S2A,B). Consistently, the levels of inflammatory cytokines, such as IFN‐γ, TNF‐α, and IL‐6, were significantly increased in the serum and lung of the mice with fluconazole pretreatment (Figure S2C,D). To further rule out that this phenomenon is lung pathogen‐specific, we used *E. coli* to infect the mice with or without fluconazole pretreatment for 3 weeks. In this model, fluconazole increased lung bacterial loads and levels of inflammatory cytokines, such as IFN‐γ, IL‐6, and TNF‐α in the serum, and IL‐1β and IL‐6 in the lung (Figure S2E–H). Although varied effects on survival rate and lung bacterial loads across different infection models were observed, these results consistently demonstrated that fluconazole‐induced dysbiosis of intestinal mycobiota aggravates lung inflammation during infection, independent of pathogen species.

Given its sensitivity to fluconazole treatment, PmCQ2‐induced lung infection was used to further assess the influence of intestinal mycobiota dysbiosis on lung inflammation during infection using amphotericin‐B (Figure 1A), another antifungal agent using a different mechanism from fluconazole [24]. We observed that the administration of amphotericin‐B replicated the increased mortality and lung bacterial loads (Figure 1K,L), as well as the enhanced production of inflammatory cytokines in the serum and lung (Figure 1M,N). Thus, these results confirmed that intestinal mycobiota perturbation using independent strategies consistently exacerbates lung inflammation and the risk of mortality during infection.

### Intestinal mycobiota is necessary to control lung inflammation during infection

**Figure 2:**
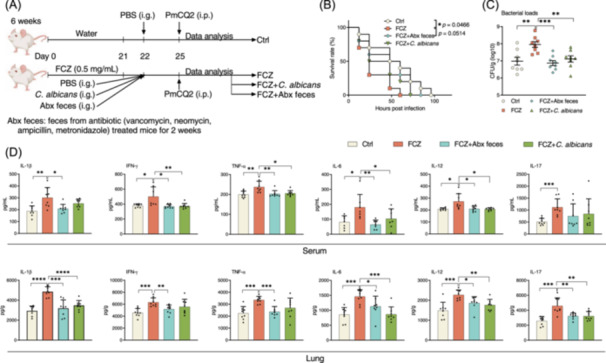
Intestinal mycobiota inoculation relieves lung inflammation during infection. (A) Experimental design for fluconazole treatment, intestinal mycobiota inoculation, and *Pasteurella multocida* type‐A (PmCQ2) infection in mice. (B) The survival rate of mice (*n* = 10). (C) Bacterial loads of mouse lung shown as mean ± SEM (*n* = 8). (D) The levels of interleukin‐1β (IL‐1β), interferon‐γ (IFN‐γ), tumor necrosis factor‐α (TNF‐α), IL‐6, IL‐12, and IL‐17 in the serum and lung (*n* = 8). Data were analyzed by Log‐rank test (B) and one‐way analysis of variance (ANOVA) (C, D) and represented as mean ± SD unless indicated. *p < 0.05, **p < 0.01, ***p < 0.001, and ****p < 0.0001.

We then asked whether recovery of intestinal mycobiota dysbiosis can rescue mice from severe lung inflammation and mortality. To reconstitute the intestinal mycobiota, we recolonized *C. albicans*, the most abundant component of intestinal mycobiota, or intestinal mycobiota into the fluconazole‐treated mice before PmCQ2 infection (Figure 2A). Intestinal mycobiota were prepared based on a previous study by collecting feces from mice treated with an antibiotic cocktail (Abx, containing vancomycin, neomycin, ampicillin, and metronidazole) [25]. Consistent with results in Figure 1, fluconazole increased the mortality, lung bacterial loads, and pro‐inflammatory cytokine levels after infection (Figure 2B–D). Notably, recolonization of intestinal mycobiota decreased the mortality, lung bacterial loads, and pro‐inflammatory cytokine production (Figure 2B–D). Recolonization of *C. albicans* also significantly reduced lung bacterial loads and partly lowered pro‐inflammatory cytokine production in the lung and serum (Figure 2B–D), suggesting that the intestinal mycobiota may be necessary to control lung inflammation during infection.

### Dysbiosis of intestinal mycobiota induces *E. coli* expansion and translocation

**Figure 3:**
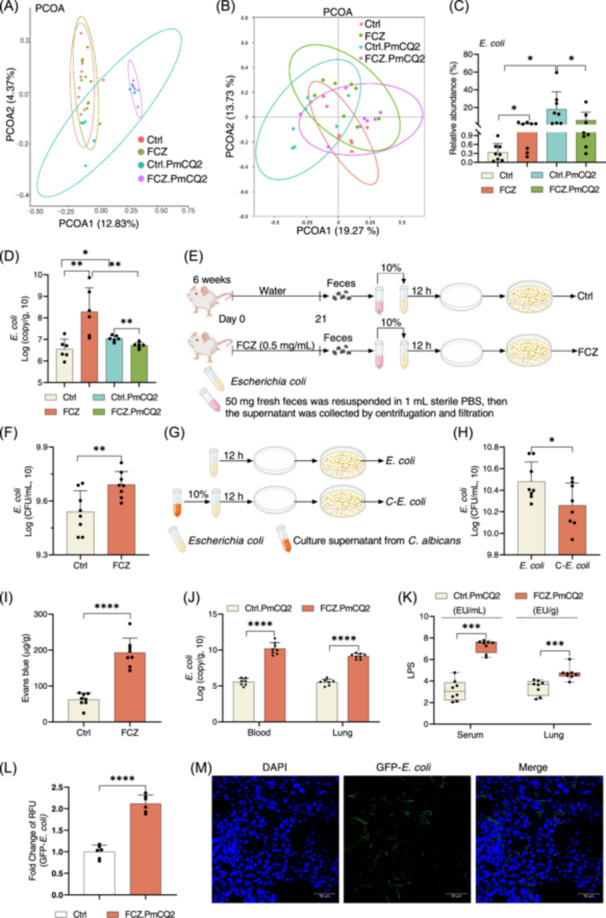
Dysbiosis of intestinal mycobiota promotes the expansion of *Escherichia coli*. (A) PCoA analysis of fungal communities in Control (Ctrl), fluconazole (FCZ), Ctrl.PmCQ2 and FCZ.PmCQ2 group (*n* = 5–10). (B) PCoA analysis of bacterial communities in Ctrl, FCZ, Ctrl.PmCQ2 and FCZ.PmCQ2 group (*n* = 8). (C) The relative abundance of *E. coli* in Ctrl, FCZ, Ctrl.PmCQ2, and FCZ.PmCQ2 group (*n* = 8). (D) Absolute abundance of the fungal population in Ctrl, FCZ, Ctrl.PmCQ2, and FCZ.PmCQ2 group (*n* = 6). (E) Experimental design for fecal metabolites from fluconazole treatment (or not) mice coculturing with *E. coli*. (F) Fecal metabolites from fluconazole treatment mice promote the growth of *E. coli* (*n* = 8). (G) Experimental design for metabolites from *Candida albicans* coculturing with *E. coli*. (H) Metabolites from *C. albicans* inhibit the growth of *E. coli* (*n* = 8). (I) Intestinal permeability assay of fluconazole treatment mice (*n* = 8). (J) Quantitative PCR for *E. coli* copies in blood and lung of Ctrl.PmCQ2 and FCZ.PmCQ2 group with data shown as mean ± SEM (*n* = 7 and 8). (K) The levels of lipopolysaccharide (LPS) in the serum and lung were analyzed by Mann–Whitney *U* test and shown as *M*(IQR) (*n* = 8). (L) The green‐fluorescent protein (GFP) labeled *E. coli* in blood (*n* = 6). (M) The GFP‐labeled *E. coli* in the lung (Scale bars, 50 μm). Data were analyzed by unpaired *t* test and represented as mean ± SD unless indicated. *p < 0.05, **p < 0.01, ***p < 0.001, and ****p < 0.0001.

To find the specific fungus associated with lung inflammation during infection, we analyzed the mouse gut fungal population following 3 weeks of treatment with fluconazole or PmCQ2 infection subsequently. PCoA plot and Venn plot showed the alteration in the composition of intestinal mycobiota among different groups (Figure 3A and Figure S3A,B,D,E,G,H). Although fluconazole decreased the richness of intestinal mycobiota (Figure 1A), no overlap in intestinal fungi from genus level was found among fluconazole versus control, control versus control.PmCQ2 and fluconazole.PmCQ2 versus control.PmCQ2 (Figure S3C,F,I).

Bacterial–fungal interactions have been proposed for shaping the development of the immune response to challenges [26], thus, we then analyzed the bacterial population. PCoA analysis showed the separation of gut microbiota among different groups (Figure 3B and Figure S4A–C), and the Venn plot exhibited the similarities and differences in bacterial population after fluconazole treatment (fluconazole vs. control), PmCQ2 infection (control vs. control.PmCQ2), or fluconazole treatment and PmCQ2 infection (fluconazole.PmCQ2 vs. control.PmCQ2) (Figure S4D–F). Several bacteria changed on species level among fluconazole versus control, control versus control.PmCQ2 or fluconazole.PmCQ2 versus control.PmCQ2 (Figure S4G–I). Notably, *E. coli* was the only bacteria from the species level overlapped among fluconazole versus control, control versus control.PmCQ2, and fluconazole.PmCQ2 versus PmCQ2 (Figure S4G–I). There was an increase in the relative abundance of intestinal *E. coli* following administration of fluconazole (fluconazole vs. control) or PmCQ2 infection (control vs. control.PmCQ2), while mice with fluconazole treatment and PmCQ2 infection (fluconazole.PmCQ2 vs. control.PmCQ2) had a lower relative abundance of intestinal *E. coli* (Figure 3C). Furthermore, the data about *E. coli* copy in the feces also supported this conclusion (Figure 3D). Considering that gut fungi affect bacterial growth, such as *Salmonella enterica* and *E. coli* [18, 19], we envisioned that intestinal mycobiota inhibits the growth of intestinal *E. coli*, thus mice with fluconazole treatment had a higher abundance of intestinal *E. coli* before PmCQ2 infection (Figure 3C,D). To test this hypothesis, the supernatant of mouse feces (with or without fluconazole treatment) was collected to coculture with *E. coli* for 12 h in vitro (Figure 3E). Similar to the observation in vivo, there was an overgrowth of *E. coli* in the fluconazole treatment group (Figure 3F). To directly demonstrate that fungi inhibit the growth of *E. coli*, we collected the culture supernatant from *C. albicans* to coculture with *E. coli* (Figure 3G). The culture supernatant of *C. albicans* inhibited the growth of *E. coli* as expected (Figure 3H).

Dysbiosis of intestinal mycobiota or the depletion of fungi destroys the intestinal barrier [27]. Thus, we hypothesized that the enhanced abundance of intestinal *E. coli* after fluconazole treatment could translocate into the lung to induce inflammation during infection by producing lipopolysaccharide (LPS), thus mice with fluconazole treatment and PmCQ2 infection have a lower abundance of intestinal *E. coli* (Figure 3C,D). To test this hypothesis, we first analyzed intestinal permeability in mice with fluconazole treatment. Fluconazole enhanced the amount of Evans blue (EB) permeation into intestine during infection (Figure 3I), indicating that fluconazole treatment destroys of intestinal barrier. We further analyzed the content of *E. coli* and LPS in the circulation and lung. As expected, the content of *E. coli* and LPS were increased in the blood and lung of fluconazole‐treated mice during infection (Figure 3J,K). In addition, to demonstrate the migration of *E. coli* actually happened, mice were colonized with a green‐fluorescent protein (GFP)‐labeled *E. coli* strain after fluconazole treatment before PmCQ2 infection. GFP‐labeled *E. coli* was detected in both the blood and lungs (Figure 3L,M). Collectively, these findings suggest that dysbiosis of intestinal fungi leads to overgrowth of *E. coli*, which translocate to the lung to aggravate lung inflammation during infection. Unfortunately, the exact metabolite produced by the fungi to inhibit growth of *E. coli* in this context is unknown.

### Dysbiosis of intestinal mycobiota induces accumulation of CD45^+^F4/80^+^Ly6G^−^Ly6C^−^CD11b^+^CD11c^+^ macrophages

**Figure 4:**
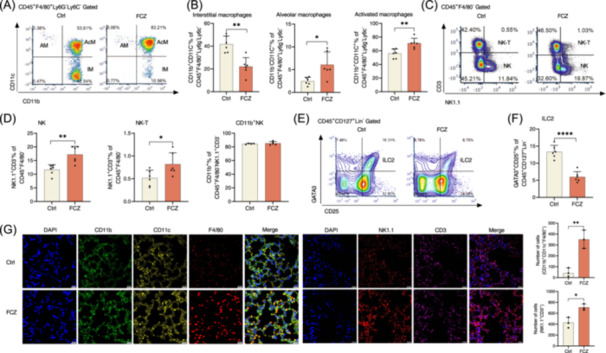
Dysbiosis of intestinal mycobiota induces accumulation of CD11b^+^CD11c^+^ macrophages. (A, B) Flow cytometry analysis of interstitial macrophages (IM, CD11b^+^CD11c^−^), alveolar macrophages (AM, CD11b^−^CD11c^+^), and activated macrophages (AcM, CD11b^+^CD11c^+^) in the lung. Interstitial macrophages, alveolar macrophages, and activated macrophages were pregated by CD45^+^F4/80^+^Ly6G^−^Ly6C^−^ (*n* = 5 and 6). (C, D) Flow cytometry analysis of NK (NK1.1^+^CD3^−^), NKT (NK1.1^+^CD3^+^), and CD11b^+^NK (CD11b^+^) cells in the lung. NK and NKT cells were pregated by CD45^+^F4/80^−^; CD11b^+^NK cells were pregated by CD45^+^F4/80^−^NK1.1^+^CD3^−^ (*n* = 4 and 6). (E, F) Flow cytometry analysis of ILC2 (GATA3^+^CD25^+^) cells in the lung. ILC2 cells were pregated by CD45^+^CD127^+^Lin^−^ (*n* = 6). (G) Immunofluorescence analysis of CD11b^+^CD11c^+^F4/80^+^ cells and NK1.1^+^CD3^+^ cells in the lung (*n* = 3, Scale bar, 20 μm). The number of CD11b^+^CD11c^+^F4/80^+^ cells was analyzed by Mann–Whitney *U* test and data shown as *M*(IQR). The data of NK1.1^+^CD3^+^ cells number was shown as mean ± SEM. Data were analyzed by unpaired *t* test and represented as mean ± SD unless indicated. *p < 0.05, **p < 0.01, and ****p < 0.0001.

To explore the underlying mechanism that dysbiosis of intestinal mycobiota aggravates lung inflammation during infection, we further analyzed changes of immune cells after fluconazole treatment and PmCQ2 infection. The immune cells in the lung were profiled using multiparametric flow cytometry (Figure S5). Fluconazole had little effect on frequencies and numbers of CD4^+^ and CD8^+^ T cells, as well as frequencies of Th1, Th2, Th17, and Treg cells (Figure S6). Although percentages of monocytes, neutrophils, and F4/80^+^ macrophages were not altered, fluconazole reduced frequency of interstitial macrophages (CD45^+^F4/80^+^Ly6G^−^Ly6C^−^CD11b^+^CD11c^−^), but increased frequencies of alveolar macrophages (CD45^+^F4/80^+^Ly6G^−^Ly6C^−^CD11b^−^CD11c^+^) and activated macrophages (CD45^+^F4/80^+^Ly6G^−^Ly6C^−^CD11b^+^CD11c^+^) (Figure 4A,B and Figure S6). In addition, fluconazole increased frequencies of NK cells (CD45^+^F4/80^−^NK1.1^+^CD3^−^) and NK‐T cells (CD45^+^F4/80^−^NK1.1^+^CD3^+^), and had no effect on frequency of CD11b^+^ NK cells (Figure 4C,D). But fluconazole reduced frequency of group 2 innate lymphoid cells (ILC2; CD45^+^CD127^+^Lin^−^GATA3^+^CD25^+^) (Figure 4E,F). Consistent with the observation using multiparametric flow cytometry, immunofluorescence analysis also showed higher number of activated macrophages (CD11b^+^CD11c^+^F4/80^+^) and NK cells (NK1.1^+^CD3^+^) in mouse lung treated with fluconazole (Figure 4G). As our previous studies had remarkably shown that the macrophages play vital roles in lung inflammation during PmCQ2 infection [21, 28, 29], therefore, we hypothesized that fluconazole‐induced mycobiota dysbiosis aggravates lung inflammation during infection through the accumulated activated macrophages in the lung.

To test this hypothesis, we depleted the macrophages in vivo by using liposome clorophosphite based on our previous methods [21]. As expected, macrophage depletion blocked the influence of fluconazole on lung inflammation during infection, such as the survival rate, lung bacterial loads, and proinflammation cytokine production in the serum and lung (Figure S7A–E). Signaling pathways, especially NLRP3 inflammasome, are related to inflammation responses, especially IL‐1β production, in macrophages [30, 31]. Thus, we used MCC950 (a specific inhibitor for NLRP3) to inhibit NLRP3 inflammasome activity in vivo. The aggravation of lung inflammation induced by fluconazole was blocked by MCC950 (Figure S7F–H). Together, these findings highlight that fluconazole enhances lung inflammation during infection through the activated macrophages. However, the roles of NK cells in this process need further investigations.

### *E. coli* promotes macrophage pro‐inflammatory responses

**Figure 5:**
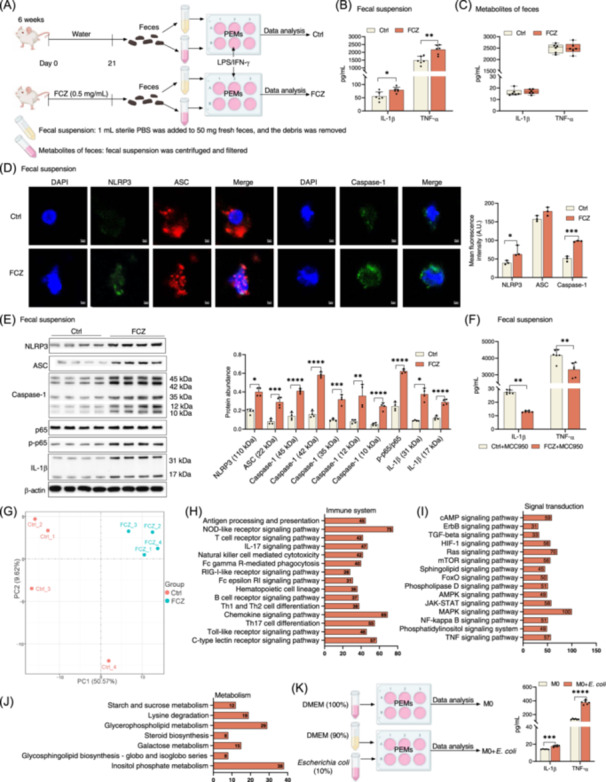
*Escherichia coli* promotes macrophage pro‐inflammatory responses. (A) Experimental design for peritoneal exudate macrophages (PEMs) polarized with lipopolysaccharide (LPS)/interferon‐γ (IFN‐γ) and cocultured with mice feces (with or without fluconazole treatment) in vitro. (B) The secretion of interleukin‐1β (IL‐1β) and tumor necrosis factor‐α (TNF‐α) from PEMs, cocultured with fecal suspension and treated with LPS/IFN‐γ (*n* = 6). (C) The secretion of IL‐1β and TNF‐α from PEMs, which cocultured with fecal metabolites and treated with LPS/IFN‐γ. The data of IL‐1β and TNF‐α were shown as *M*(IQR) while the data of IL‐1β was analyzed by Mann–Whitney *U* test (*n* = 6). (D) Confocal microscopy analysis of PEMs, cocultured with fecal suspension and treated with LPS/IFN‐γ, by immunostaining for NLRP3 (green), ASC (red), and Caspase‐1 (green) (*n* = 3; Scale bars, 2 μm). Data were shown as *M*(IQR). The mean fluorescence intensity (A.U.) of NLRP3 and ASC were analyzed by Mann–Whitney *U* test. (E) Protein abundance of NLRP3, ASC, Caspase‐1, p65, p‐p65, and IL‐1β in PEMs, cocultured with fecal suspension and treated with LPS/IFN‐γ (*n* = 4). (F) IL‐1β and TNF‐α secretion from PEMs, cocultured with fecal suspension and treated with LPS/IFN‐γ and MCC950 (20 μM) (*n* = 6). (G) PCA analysis of PEMs, cocultured with fecal suspension and treated with LPS/IFN‐γ (*n* = 4). (H, J) The KEGG analysis of differently expressed genes from G associating with the immune system (H), signal transduction (I), and metabolism pathway (J), Fold Change > 1; *Padj* < 0.05. (K) The IL‐1β and TNF‐α secretion from PEMs, which cocultured with *E. coli* (*n* = 5 and 6). Data were analyzed by unpaired *t* test and represented as mean ± SD unless indicated. *p < 0.05, **p < 0.01, ***p < 0.001, and ****p < 0.0001.

To further explore the underlying mechanism of fluconazole‐induced dysbiosis of intestinal mycobiota on macrophage activation, we activated peritoneal exudate macrophages (PEMs) based on our previous methods [21, 31] and cocultured with fecal suspension (contain bacteria) or metabolites (the supernatant of fecal suspension, free of bacteria) from fluconazole‐treated mice (Figure 5A). Interestingly, the fecal suspension, rather than fecal metabolites, from fluconazole‐treated mice increased the production of TNF‐α and IL‐1β (makers for M1 macrophage activation [31, 32]) from PEMs (Figure 5B,C). Furthermore, fecal suspension from fluconazole‐treated mice highly enhanced activation of NLRP3 inflammasome and NF‐kappa B in PEMs (Figure 5D,E). Similar to the results in vivo, MCC950 blocked the fecal suspension‐induce production of IL‐1β and TNF‐α in PEMs (Figure 5F). In addition, RNA‐seq analysis also revealed that fecal suspension from fluconazole‐treated mice induced changes in PEMs involving inflammation‐associated signaling pathways, such as “mTOR signaling pathway,” “NF‐kappa B signaling pathway,” and “NOD‐like receptor signaling pathway” (Figure 5G−J and Figure S8).

Based on the above results that fluconazole induces outgrowth of intestinal *E. coli* (Figure 3C,D), and that the fecal suspension from fluconazole‐treated mice activates macrophages (Figure 5B–E), we hypothesized that it is the *E. coli* that activate macrophages and is responsible for the excessive lung inflammation after fluconazole treatment during infection. Notably, *E. coli* alone could increase IL‐1β and TNF‐α production from naive PEMs in vitro (Figure 5K).

### TLR4 deficiency attenuates lung inflammation induced by mycobiota dysbiosis

**Figure 6:**
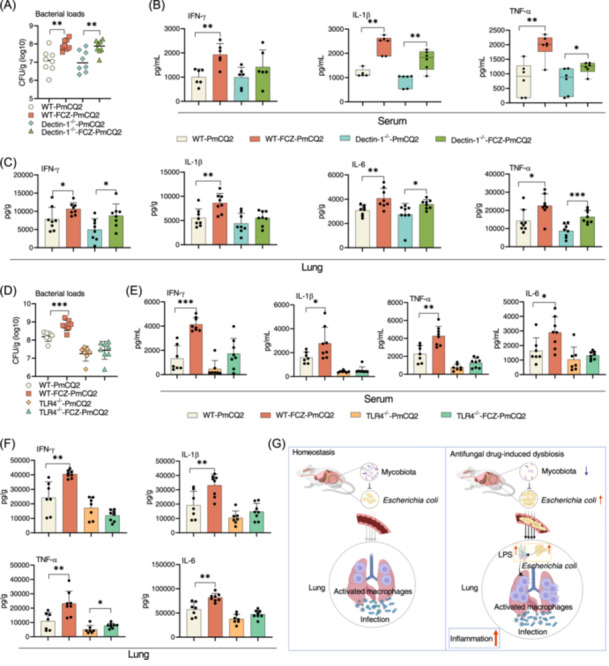
TRL4 deficiency attenuates lung inflammation. (A) Bacterial burdens of mouse lung with data shown as mean ± SEM (*n* = 8). (B) The levels of interferon‐γ (IFN‐γ), interleukin‐1β (IL‐1β), and tumor necrosis factor‐α (TNF‐α) in the serum. The data of TNF‐α and IL‐1β were analyzed by Mann–Whitney *U* test and shown as *M*(IQR) (*n* = 5 and 6). (C) The levels of IFN‐γ, IL‐1β, IL‐6, and TNF‐α in the lung (*n* = 8). (D) Bacterial burdens of mouse lung with data shown as mean ± SEM (*n* = 7 and 8). (E, F) The levels of IFN‐γ, IL‐1β, TNF‐α, and IL‐6 in the serum and lung (*n* = 7 and 8). (G) Antifungal drug‐induced dysbiosis of intestinal mycobiota induces intestinal *Escherichia coli* over‐expansion and migration into the blood and lung, where activates the CD45^+^F4/80^+^Ly6G^−^Ly6C^−^CD11b^+^CD11c^+^ macrophages, resulting in aggravation of lung inflammation during infection, created with BioRender.com. Data were analyzed by unpaired *t* test and represented as mean ± SD unless indicated. *p < 0.05, **p < 0.01, and ***p < 0.001.

LPS activates macrophage TLR4 to produce pro‐inflammatory cytokines [33]. To further verify that it is *E. coli*/LPS rather than fungi after fluconazole treatment to aggravate lung inflammation during infection, we treated wild‐type C57BL/6, Dectin‐1(recognition of beta‐1,3/1,6 glucans on fungi)^−/−^ (Figure S9A), or TLR4 (recognition of LPS)^−/−^ (Figure S9B) mice (no change for the abundance of intestinal *E. coli* [34]) with fluconazole before PmCQ2 infection. In wild‐type C57BL/6 mice, fluconazole aggravated lung inflammation during infection based on higher lung bacterial loads and pro‐inflammatory cytokine production (Figure 6A–C). In Dectin‐1^−/−^ mice, fluconazole still increased the lung bacterial loads and production of IL‐1β and TNF‐α in the serum as well as IFN‐γ, IL‐6, and TNF‐α in the lung (Figure 6A–C). However, fluconazole had no effect on the lung bacterial loads and production of pro‐inflammatory cytokines in the serum and lung in TLR4^−/−^ mice (Figure 6D–F), suggesting that the translocated *E. coli* depends on the TLR4 to activate inflammation. Collectively, *E. coli*/LPS after fluconazole treatment aggravates lung inflammation during infection.

## DISCUSSION

In this study, we found that dysbiosis of gut mycobiota led to gut *E. coli* overexpansion and migration to the lung during infection. Mechanistically, *E. coli* activates the TLR4 signaling in CD45^+^F4/80^+^Ly6G^−^Ly6C^−^CD11b^+^CD11c^+^ macrophages, resulting in aggravation of lung inflammation during infection. Previous investigation has also suggested that fungi control the growth of bacteria. For example, *M. circinelloides* induces an increase in the genus *Bacteroides* and a decrease in *Akkermansia* [16], and *C. albicans* reduces colonization of probiotic *Lactobacillus* strains in the gut [35]. Notably, the underlying mechanism that fungi affect the colonization, assembly, growth, and function of bacteria includes 1) secreting some small molecules, such as phosphatase, β‐1,2‐mannans, ethanol, and antimicrobial peptides; 2) producing quorum sensing compounds like farnesol; 3) regulating the bacterial growth conditions, such as pH and oxygen content [13, 18, 36, 37]. However, whether gut mycobiota affects *E. coli* growth through these mechanisms in this study requires further investigation. In addition, except for *E. coli*, intestinal mycobiota clearance leads to diverse changes in the bacterial community, such as a lower abundance of *Lactobacillus* in the intestine. Accumulating evidence has shown that gut bacterial dysbiosis occurs in various acute or chronic lung inflammation diseases such as idiopathic pulmonary fibrosis, asthma, chronic obstructive pulmonary disease, and lung cancers [38, 39, 40]. Fecal microbiota transplantation from a healthy counterpart or depletion of microbiota by antibiotics treatment or germ‐free mice can alleviate lung inflammation in these disease models. Gut microbiota releases various metabolites into the blood, which influences host lung immunity and function [41]. Also, gut microbiota imprints intestinal immune cells such as DCs and T cells, which can migrate into the lung to influence lung immune response in healthy and disease situations [42]. Thus, it is interesting to explore the mechanisms by which fungi affect the changes in these bacterial communities. Also, the physiological roles of these altered intestinal bacteria in the intestinal mycobiota‐mediated gut–lung axis need further exploration.

Interestingly, we found that the crosstalk between gut mycobiota and bacteria aggravates lung inflammation during infection. Previous studies have found that intestinal fungi or bacteria shape intestinal and even extraintestinal diseases. For instance, the administration of antifungal drugs exacerbates colitis [17]. Gut mycobiota regulate host susceptibility to ovalbumin (OVA)‐induced allergic airway inflammation [43]. Although previous compelling investigation has shown that intestinal fungi–bacteria crosstalk affect the progression of diseases, like alcoholic hepatitis [44]. However, the underlying mechanisms by which the interaction between intestinal fungi and bacteria shapes intestinal or extraintestinal diseases are uncovered. Here, we found that intestinal mycobiota dysbiosis leads to the expansion of intestinal *E. coli* and migration to the lung, which enhances lung inflammation during infection. This result shows that intestinal fungi–bacteria crosstalk shapes lung inflammation during infection through the gut–lung axis. However, the exact mechanisms by which the intestinal fungi–bacteria crosstalk dictate the pathogenesis or progression of intestinal diseases or other extraintestinal diseases need further exploration.

The gut microbiota is an intricate microbial community. In addition to the interaction between fungi and bacteria, other microbes seem to interact with each other and influence host health. Studies have found that intestinal bacteria promote infection of several mammalian enteric RNA viruses by increasing viral fitness [45]. Thus, the interaction among these intestinal inhabitants on host health needs further well‐designed investigations. In addition, host commensal microbiota is distributed into the skin, oral cavity, gastrointestinal tract, respiratory tract, and lower genital tract. Although the gastrointestinal tract is one of the most well‐studied sites for examining interactions between fungi and the resident bacteria, fungal–bacterial interactions in extraintestinal sites also determine host health [46]. In the oral cavity, *C. albicans* enhances the virulence of *S. aureus*, which may cause serious nosocomial and community‐acquired infections [47]. Furthermore, the interaction between *C. albicans* and *Pseudomonas aeruginosa* in the respiratory tract also regulates the lung function of patients with cystic fibrosis [48]. Microbial interactions among different sites may differ, thus their effects on different diseases may be context‐dependent. Here, we only described the role of intestinal fungi–bacteria crosstalk in lung inflammation during infection; the influence of extraintestinal fungi–bacteria crosstalk on regulating diseases requires more investigation.

## CONCLUSION

Collectively, dysbiosis of the intestinal mycobiota by antifungal drugs aggravates the severity of lung inflammation caused by bacterial infection. Antifungal drug treatment results in the expansion of intestinal *E. coli* and even migration to blood and lungs during infection. *E. coli* activates the TLR4 signaling in CD45^+^F4/80^+^Ly6G^−^Ly6C^−^CD11b^+^CD11c^+^ macrophages, resulting in aggravation of lung inflammation during infection (Figure 6G). However, there are various open questions in this field. For example, the exact mechanism by which intestinal mycobiota inhibits intestinal *E. coli* and the underlying mechanism by which dysbiosis of the intestinal mycobiota destroys the intestinal mucosal barrier remain to be explored.

## METHODS

### Bacterial strains

Based on our previous reports [22, 49], PmCQ2, *S. pneumonia, E. coli, and C. albicans* (SC5314) were cultivated in Martin's broth including 5% horse serum, THB broth, LB broth, and Yeast extract peptone dextrose broth, respectively.

### Mice

All experiments were performed using 6‐ to 8‐week‐old mice, which were age‐ and gender‐matched for individual experiments. Kunming (KM) mice used in Figures 1, 2, 3 and Figures S1–4, S7 were purchased from the Laboratory Animal Center of Third Military Medical University (Chongqing, China). Institute of Cancer Research (ICR) mice used in Figures 3, 4, 5 and Figure S6, wild‐type C57BL/6 and TLR4^−/−^ mice used in Figure 6 and Figure S9 were purchased from Gempharmatech Co., Ltd. Dectin‐1^−/−^ mice used in Figure 6 and Figure S9 were generously provided by Prof. Jie Yin (Hunan Agricultural University, Changsha, China).

### Murine primary PEMs

Murine primary PEMs were obtained as referred to in our previous study [21]. Briefly, 6‐ to 8‐week‐old ICR mice were injected with 4% thioglycolate for 2 to 4 days. PEMs retrieved from the murine peritoneal cavity were flushed with DMEM and cultured (complete DMEM) for adherence over a period of 4 h. LPS (1 μg/mL) plus IFN‐γ (20 ng/mL) were added for PEMs pro‐inflammatory polarization.

### Antifungal and antibiotic treatments

For antifungal experiments, mice were treated with fluconazole (0.5 mg/mL, MB1288; Meilunbio) or amphotericin‐B (0.1 mg/mL, MB1013; Meilunbio) in their drinking water for 3 weeks based on a previous report [24]. For antibiotic experiments, an antibiotic cocktail in ddH_2_O, containing ampicillin (1 mg/mL, MB1507; Meilunbio), metronidazole (1 mg/mL, MB2200; Meilunbio), neomycin (1 mg/mL, MB1716; Meilunbio), and vancomycin (0.5 mg/mL, MB1260; Meilunbio), was added to the drinking water of mouse for 14 days.

### In vivo pathogenic bacteria infection

Mice were intraperitoneally infected with PmCQ2, *S. pneumoniae*, and *E. coli* at a dose of 2.2 × 10^5^ CFU diluted in PBS. Serum and lung samples were collected 12 h after mouse infection. Survival rates were monitored after the mice were infected.

### PEMs coculture assay

The feces (around 50 mg), from fluconazole‐treated mice, were homogenized and resuspended in 1 mL sterile PBS. The homogenate of feces‐filtered residue was obtained from the fecal suspension, which was centrifuged (3000 rpm, 10 min) and filtered with a 0.22 μm filter to get fecal metabolites. PEMs were cocultured with fecal suspension and metabolites and treated with LPS/IFN‐γ for 6 h for M1 polarization. *E. coli* was cultured in LB broth for 12 h, then PEMs were cocultured with *E. coli* for 6 h. Culture supernatant and cells were collected for further analysis.

### Fungi colonization treatment

Mice, treated with fluconazole, were inoculated with 200 μL of *C. albicans* (SC5314, 1 × 10^7^ CFU in PBS, i.g.) and intestinal mycobiota (Abx‐treated mice fecal suspension, 50 mg/mL) for 3 days. Then, mice were infected with PmCQ2 (i.p.). Serum and lung samples were collected 12 h after mice infection.

### Deletion of macrophages in vivo

Macrophage deletion was executed as described previously [21]. Briefly, mice with or without fluconazole treatment were intraperitoneally administered 200 μL liposome clorophosphite (LIPOSOMA) to induce macrophage apoptosis, then mice were infected with PmCQ2 after 48 h of macrophage deletion. Serum and lung samples were collected 12 h after the mice infection.

### In vivo MCC950 treatment

Mice, with or without fluconazole treatment, were intraperitoneally injected with MCC950 (an inhibitor for NLRP3 inflammation; 20 mg/kg body weight) and infected PmCQ2 at the same time. Serum and lung samples were collected 12 h after mice infection.

### Histological analysis of lung

The lung samples of mice were immersed in 4% paraformaldehyde (PFA) at 4°C overnight for fixation. Fixed samples were dehydrated in graded ethanol and embedded in paraffin. Then, the paraffin samples were cut into sections (5 μm), which were stained with hematoxylin and eosin.

### Count of bacteria CFUs

Bacteria CFUs in mouse lungs were counted as previously reported [29]. Briefly, mouse lungs were homogenized in PBS and diluted to appropriate concentrations. 100 μL solution was inoculated on Martin's broth agar, LB broth agar, or THB broth agar and incubated at 37°C for 24 h. Then, colony numbers were counted. Bacterial CFUs were normalized to the weight of the lungs. For the count of *E. coli* culture in vitro, *E. coli* and fecal metabolites or *C. albicans* culture supernatant were cocultured in 37°C for 12 h. One hundred microliter solution of appropriate concentration was inoculated on eosin‐methylene blue agar. After incubation at 37°C for 24 h, colony numbers were counted.

### Enzyme‐linked immunosorbent assay (ELISA)

Inflammatory cytokines including IL‐1β, TNF‐α, IL‐6, IL‐12, IFN‐γ, and IL‐17 in the serum and lung lysates of mice, and IL‐1β and TNF‐α in the culture supernatants of cells were measured by commercial kits according to the instruction.

### RT‐qPCR analysis

Total RNA from the lung was extracted using Tissue RNA Purification Kit PLUS (EZBioscience) and quantified using Nanodrop 2000. It was reverse‐transcribed to cDNA using Color Reverse Transcription Kit (EZBioscience) and then mixed with SYBR Green (EZBioscience) for RT‐qPCR analysis on the Quant Studio 6 Real‐Time PCR System (Thermo Fisher Scientific). The data were counted through the 2^−ΔΔCt^ method using β‐actin for normalization. Genomic DNA was extracted from lung, blood, and feces in accordance with the protocol of Biospin whole blood/cell/tissue genomic DNA Extraction kit (BIOER) or TIANamp stool DNA kit (Tiangen), and quantified with Nanodrop 2000. RT‐qPCR was performed using SuperReal PreMix (Probe) (Tiangen) on the Quant Studio 6 Real‐Time PCR System (Thermo Fisher Scientific). Taking each gram tissue as the detection unit, the copy of *E. coli* was calculated according to the CT value and standard curve, and the copy of *E. coli* was converted into logarithm statistical analysis. The primers and probes are listed in Table S1.

### Immunofluorescence

For cell immunofluorescence staining, cells were immersed in 4% PFA for 30 min at room temperature for fixation and blocked with QuickBlock™ Blocking Buffer (Beyotime) for 30 min at room temperature. Then, cells were incubated with primary antibodies at 4°C overnight and secondary antibodies for 1 h at room temperature. Finally, cells were stained with DAPI (Beyotime), mounted, and viewed using a confocal fluorescence microscope (Zeiss). For tissue immunofluorescence staining, fresh lungs were fixed with 4% PFA for 24 h and embedded in paraffin, which were cut into sections. Tissue sections were washed sequentially in xylene and graded ethanol solutions and then hydrated in ddH2O, and were boiled in citrated buffer for 10 min for antigen retrieval. Tissue sections were blocked with 5% bovine serum albumin (BSA) at 4°C overnight, incubated with primary and secondary antibodies, stained using DAPI, mounted, and viewed consistent with cell immunofluorescence staining.

### Flow cytometry

Mouse lungs were used to prepare single‐cell suspension and stain with Live/dead kit distinguishing live or dead cells. Cells were stained with the surface marker of CD45 and selected CD45^+^ cells for the following experiments. CD45^+^ cells were stained with T cell surface markers of CD3, CD4, CD8; or NK cell and Macrophage surface markers of F4/80, CD3, NK1.1, Ly6G, Ly6C, CD11c, CD11b; or ILC surface markers of CD127, Lin, CD25. Then cells were either fixed or cell stained with T cell intracellular markers of T‐bet, RORγt, FoxP3, GATA3, or ILC intracellular marker of GATA3. All antibodies are listed in the Table S1 Key resource table. Flow cytometry was performed on an LSRFortessa (BD Biosciences) and data were analyzed using the FlowJo Software.

### RNA‐Seq analysis

NovoGene conducted RNA extraction, verified quality, prepared libraries, and performed sequencing. Simply, clean data were obtained by filtering low‐quality reads from raw data and mapped to the reference genome. Differential expression analysis was carried out using DESeq2. GO, and KEGG pathway enrichment analysis of the DEGs (Fold Change > 1; *Padj* < 0.05) were performed.

### Western blot analysis

The lung and cell samples were lysed with RIPA Lysis Buffer (Beyotime). Protein concentration was determined using the BCA protein assay kit (Beyotime), and an equal amount of protein was loaded for sodium dodecyl sulphate‐polyacrylamide gel electrophoresis. Then, proteins were transferred from the gel to polyvinylidene difluoride membranes. Membranes were blocked with 5% nonfat powdered milk (Beyotime), incubated with primary and secondary antibodies, and visualized using a chemiluminescent reagent. Quantification of signal optical density on the film was quantified by ImageJ software.

### Intestinal permeability assay

An intestinal permeability assay was executed as described previously [50]. Briefly, mice were treated with or without fluconazole. The small intestine (6 cm) was collected and used as a sac by ligating both ends. Then, 200 μL 1.5% (w/v) EB was injected into the sac, and the sac was incubated in 20 mL Krebs buffer for 30 min. The intestinal lumen was irrigated with saline until the rinsing solution was clear. The intestines were weighed after drying at 37°C for 24 h and incubated with formamide at 50°C for 24 h. Finally, the permeability of the intestine was assessed using EB via a microplate reader (655 nm), and the amount of EB was calculated according to the standard curve. The amount of EB per gram was calculated to represent intestinal permeability.

### Quantification of the absolute abundance of the fungal populations

Quantification of the absolute abundance of the fungal population was executed as described previously [51]. Briefly, genomic DNA was extracted from feces with the protocol of TIANamp stool DNA kit (Tiangen) and quantified with Nanodrop 2000. RT‐qPCR was performed using Femto™ Fungal DNA Quantification Kits (Zymo Research) according to the manufacturer's protocols. Results were expressed as fungal genome copy number/g feces.

### *E. coli* colonization treatment

Mice, treated with fluconazole, were inoculated with 200 μL of GFP‐labeled *E. coli* strain (1 × 10^8^ CFU in PBS, i.g.) for 7 days. Then, mice were infected with PmCQ2 (i.p.). Blood and lung samples were collected 12 h after mice infection. The GFP‐labeled *E. coli* blood was detected by a microplate reader (Ex 475 nm, Em 509 nm), and the result was expressed as fold change relative to the RFU of control mice (without fluconazole treatment) blood. Lung samples were embedded in an optimal cutting temperature compound (OTC). Then, cryostat section was performed. Tissue sections were stained, mounted, and viewed using a confocal fluorescence microscope (Zeiss).

### Fungal ITS1 sequencing

Fungal DNA was extracted using the QIAamp DNA Stool Mini Kit (Qiagen) and served as the template for PCR amplification of fungal ITS genes using barcoded primers and Takara Ex Taq (Takara). The ITS1 variable regions of ITS genes were amplified with the universal primers ITS1F (5′‐CTTGGTCATTTAGAGGAAGTAA‐3′) and ITS2 (5′‐GCTGCGTTCTTCATCGATGC‐3′). The PCR products were cleaned up with AMPure XP beads (Beckman), and their concentrations were adjusted accordingly for sequencing. The sequencing process was carried out on an Illumina NovaSeq. 6000 with 250 bp paired‐end reads (Illumina Inc.; OE Biotech Company).

Library sequencing and data processing were performed by OE Biotech Co., Ltd. Paired‐end reads underwent preprocessing using Trimmomatic software and paired‐end reads were assembled using FLASH software. Subsequently, sequences underwent further denoising. Clean reads underwent primer sequence removal and clustering to generate operational taxonomic units (OTUs) with a 97% similarity cutoff using Vsearch software. A representative read for each OTU was chosen with the QIIME package. All representative reads were annotated and blasted against the Unite database using BLAST.

### Bacterial 16s rDNA sequencing

16S rDNA sequencing and analysis were counted as previously reported [52]. Briefly, DNA was extracted using QIAamp DNA Stool Mini Kit (Qiagen, Germany) and the ribosomal 16S rDNA V4 region was amplified using universal primers 515F (5′‐GTGCCAGCMGCCGCGGTAA‐3′) and 806R (5′‐GGACTACHVGGGTWTCTAAT‐3′). PCR products were purified using Universal DNA Purification Kit (Tiangen), and sequencing libraries were generated using NEB Next® Ultra™ II FS DNA PCR‐free Library Prep Kit (NEB). The libraries were quantified and sequenced on Illumina platforms. Paired‐end reads were merged, and quality filtering was done to obtain high‐quality Clean Tags. The tags were compared with the Silva database using the UCHIME Algorithm to detect chimera sequences. Effective tags were then analyzed and assigned to operational taxonomic units (OTUs) with ≥97% similarity using Uparse software. Representative sequences from each OTU were annotated to species level with the Mothur method and the SSUrRNA database of Silva138. All the sequencing and analysis were counted by Novogene, Co., Ltd.

### Statistical analysis

Statistical analyses were carried out in GraphPad Prism 8.0 (GraphPad Software), and all data were represented as mean ± SD, mean ± SEM, or median with interquartile range (*M*(IQR)). The survival rate of mice was evaluated using Kaplan–Meier analysis. Data between two groups were calculated by unpaired *t* test if the data followed a normal distribution and had equal variance, by unpaired *t*‐test with Welch's correction if the data followed a normal distribution but with unequal variance, or by Mann–Whitney *U* test if the data were not Normal distribution. Multigroup was analyzed by one‐way ANOVA followed by Dunnett multiple comparisons if the data followed a normal distribution and had equal variance, or else calculated by Kruskal–Wallis followed by Bonferroni multiple comparisons. *p*＜0.05 was defined as a significant difference; data in the figures are presented as *p < 0.05, **p < 0.01, ***p < 0.001, and ****p < 0.0001.

## AUTHOR CONTRIBUTIONS

Wenkai Ren designed the experiment. Youxia Wang and Fang He conducted the experiments. Youxia Wang, Ziyi Han, and Bingnan Liu analyzed the data and drafted the manuscript. Xiaoyan Wu, Xuefei Wang, and Yuexia Liao conducted flow cytometry. Jielin Duan assisted with experiments. Youxia Wang and Ziyi Han prepared the figures. Wenkai Ren revised the manuscript. All authors have read the final manuscript and approved it for publication.

## CONFLICT OF INTEREST STATEMENT

The authors declare no conflict of interest.

## ETHICS STATEMENT

The ethics application was approved by the Research Ethics Committee of Southwest University (No. IACUC‐20201020‐03) and the South China Agricultural University (No. 2024f326).

## Supporting information


**Figure S1:** Relative mRNA expressions of lung inflammatory cytokines.
**Figure S2:** Dysbiosis of intestinal mycobiota aggravates lung inflammation during infection.
**Figure S3:** Fluconazole treatment affects intestinal mycobiota.
**Figure S4:** Fluconazole treatment affects intestinal bacteria.
**Figure S5:** Gate strategy of flow cytometry.
**Figure S6:** Effect of intestinal mycobiota on lung immune cells.
**Figure S7:** Deletion of macrophages relieves lung inflammation during infection.
**Figure S8:** *E. coli* activates the immune response of macrophages.
**Figure S9:** Western blots analysis of Dectin‐1 and TLR4.

**Table S1:** Key resource table.

## Data Availability

Raw RNA sequencing data have been deposited at NCBI Sequence Read Archive (https://www.ncbi.nlm.nih.gov/sra) under accession number PRJNA1037186. Raw 16S rDNA sequencing data have been deposited at NCBI Sequence Read Archive (https://www.ncbi.nlm.nih.gov/sra) under accession number PRJNA1036781. Raw ITS rDNA sequencing data have been deposited at NCBI Sequence Read Archive (https://www.ncbi.nlm.nih.gov/sra) under accession number PRJNA1036787. Supplementary materials (figures, tables, graphical abstract, slides, videos, Chinese translated version, and update materials) may be found in the online DOI or iMeta Science http://www.imeta.science/. Research data are not shared.
