# Supplementary material for: Interaction between intestinal mycobiota and microbiota shapes lung inflammation
Source: Imeta. 2024 Sep 14;3(5):e241. doi: 10.1002/imt2.241 (PMC11487552; doi:10.1002/imt2.241)
Supplement: Supplementary file 1 — Figure S1: Relative mRNA expressions of lung inflammatory cytokines. Figure S2: Dysbiosis of intestinal mycobiota aggravates lung inflammation during infection. Figure S3: Fluconazole treatment affects intestinal mycobiota. Figure S4: Fluconazole treatment affects intestinal bacteria. Figure S5: Gate strategy of flow cytometry. Figure S6: Effect of intestinal mycobiota on lung immune cells. Figure S7: Deletion of macrophages relieves lung inflammation during infection. Figure S8: E. coli activates the immune response of macrophages. Figure S9: Western blots analysis of Dectin‐1 and TLR4. [file IMT2-3-e241-s002.docx]

**Supporting information to Interaction between intestinal mycobiota and microbiota shapes** **lung inflammation**

**Running title: Gut homeostasis shapes lung inflammation**

Youxia Wang^1#^, Fang He^2#^, Bingnan Liu^1#^, Xiaoyan Wu^1^, Ziyi Han^1^, Xuefei Wang^3^, Yuexia Liao^4^, Jielin Duan^5^, Wenkai Ren^1*^

^1^State Key Laboratory of Swine and Poultry Breeding Industry, College of Animal Science, South China Agricultural University, Guangzhou 510642, China

^2^College of Veterinary Medicine, Southwest University, Chongqing 400715, China

^3^School of Basic Medical Sciences, Capital Medical University, Beijing 100069, China

^4^School of Nursing & School of Public Health, Yangzhou University, Yangzhou 225009, China

^5^Department of Allergy and Clinical Immunology, State Key Laboratory of Respiratory Disease, National Clinical Research Center for Respiratory Disease, Guangzhou Institute of Respiratory Health, the First Affiliated Hospital of Guangzhou Medical University, Guangzhou 510642, China

^#^These authors contributed equally: Youxia Wang, Fang He, Bingnan Liu

*Correspondence: [renwenkai19@scau.edu.cn](mailto:renwenkai19@scau.edu.cn) (Wenkai Ren)


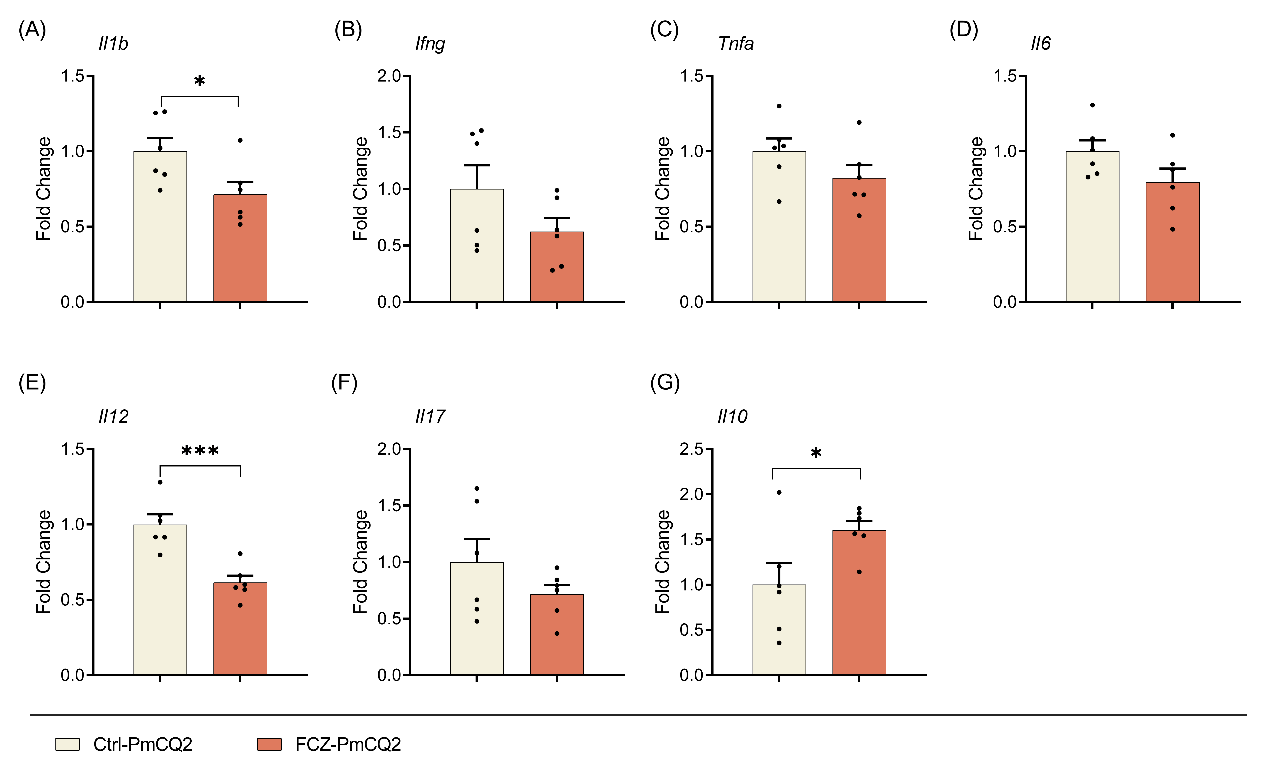


**Figure S1** Relative mRNA expressions of lung inflammatory cytokines. (A-G) Relative mRNA expressions of (A) *Il1b*, (B) *Ifng*, (C) *Tnfa*, (D) *Il6*, (E) *Il12*, (F) *Il17*, and (G) *Il10* in the lung (*n* = 6). Data were analyzed by unpaired *t* test and represented as mean ± SEM unless indicated. **p* < 0.05, ***p* < 0.01, ****p* < 0.001, and *****p* < 0.0001.


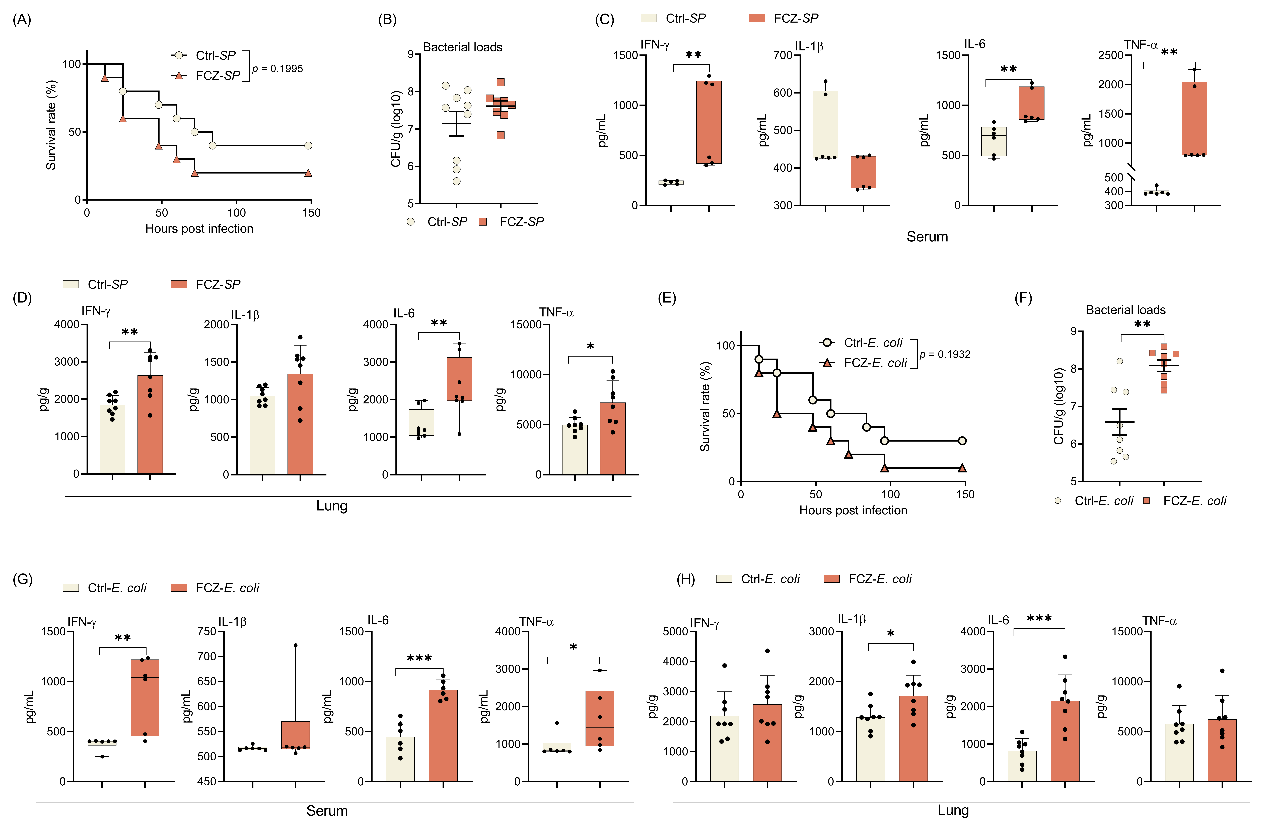


**Figure S2** Dysbiosis of intestinal mycobiota aggravates lung inflammation during infection. (A) The survival rate of mice with the data analyzed by Log-rank test (*n* = 10). (B) Bacterial burdens of mouse lung with data shown as mean ± SEM (*n* = 8 and 9). (C) The levels of IL-1β, IFN-γ, TNF-α, and IL-6 in the serum with the data analyzed by Mann-Whitney *U* test and shown as M(IQR) (*n* = 5-6). (D) The levels of IL-1β, IFN-γ, TNF-α, and IL-6 in the lung with the data of IL-6 analyzed by Mann-Whitney *U* test and shown as M(IQR) (*n* = 8). (E) The survival rate of mice with the data analyzed by Log-rank test (*n* = 10). (F) Bacterial burdens of mouse lung with data shown as mean ± SEM (*n* = 8). (G) The levels of IL-1β, IFN-γ, TNF-α, and IL-6 in the serum. The data of IL-1β, TNF-α and IFN-γ were analyzed by Mann-Whitney *U* test and shown as M(IQR) (*n* = 6). (H) The levels of IL-1β, IFN-γ, TNF-α, and IL-6 in the lung (*n* = 8). Mice in A-D were given (or not) fluconazole in their drinking water for 21 days before *S. pneumoniae* infection, while mice in E-H were given (or not) fluconazole in their drinking water for 21 days before *E. coli* infection. Data were analyzed by unpaired *t* test and represented as mean ± SD unless indicated. **p* < 0.05, ***p* < 0.01, ****p* < 0.001, and *****p* < 0.0001.


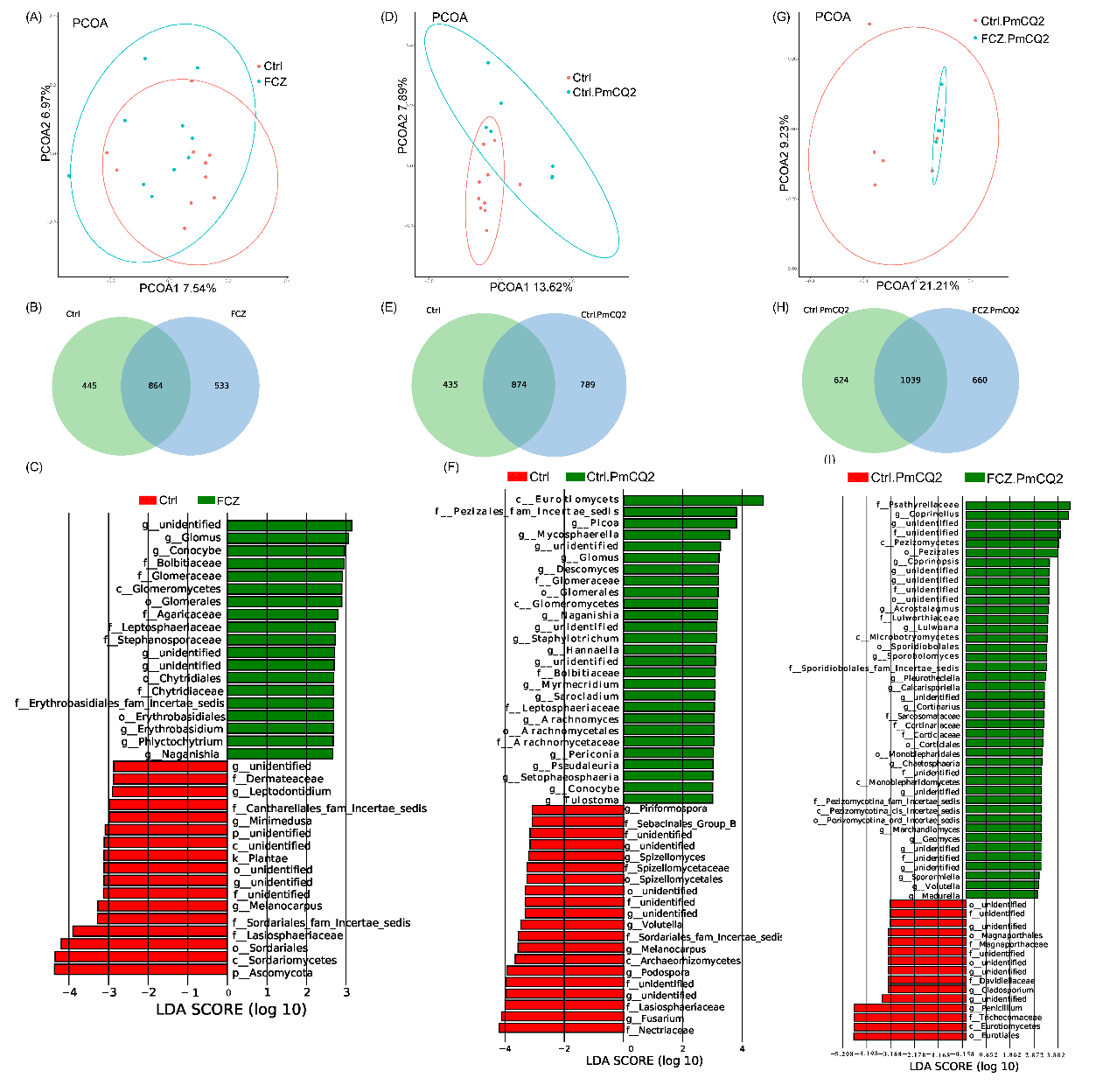


**Figure S3** Fluconazole treatment affects intestinal mycobiota. (A, D, G) PCoA analysis, Venn analysis and LEfSe analysis of fungal communities with fluconazole treatment (LDA Score ≥ 3; *n* = 10). (B, E, H) PCoA analysis, Venn analysis and LEfSe analysis of fungal communities with PmCQ2 infection (LDA Score ≥ 3; *n* = 7 and 10). (C, F, I) PCoA analysis, Venn analysis and LEfSe analysis of fungal communities with fluconazole treatment and PmCQ2 infection (LDA Score ≥ 3; *n* = 5 and 7).


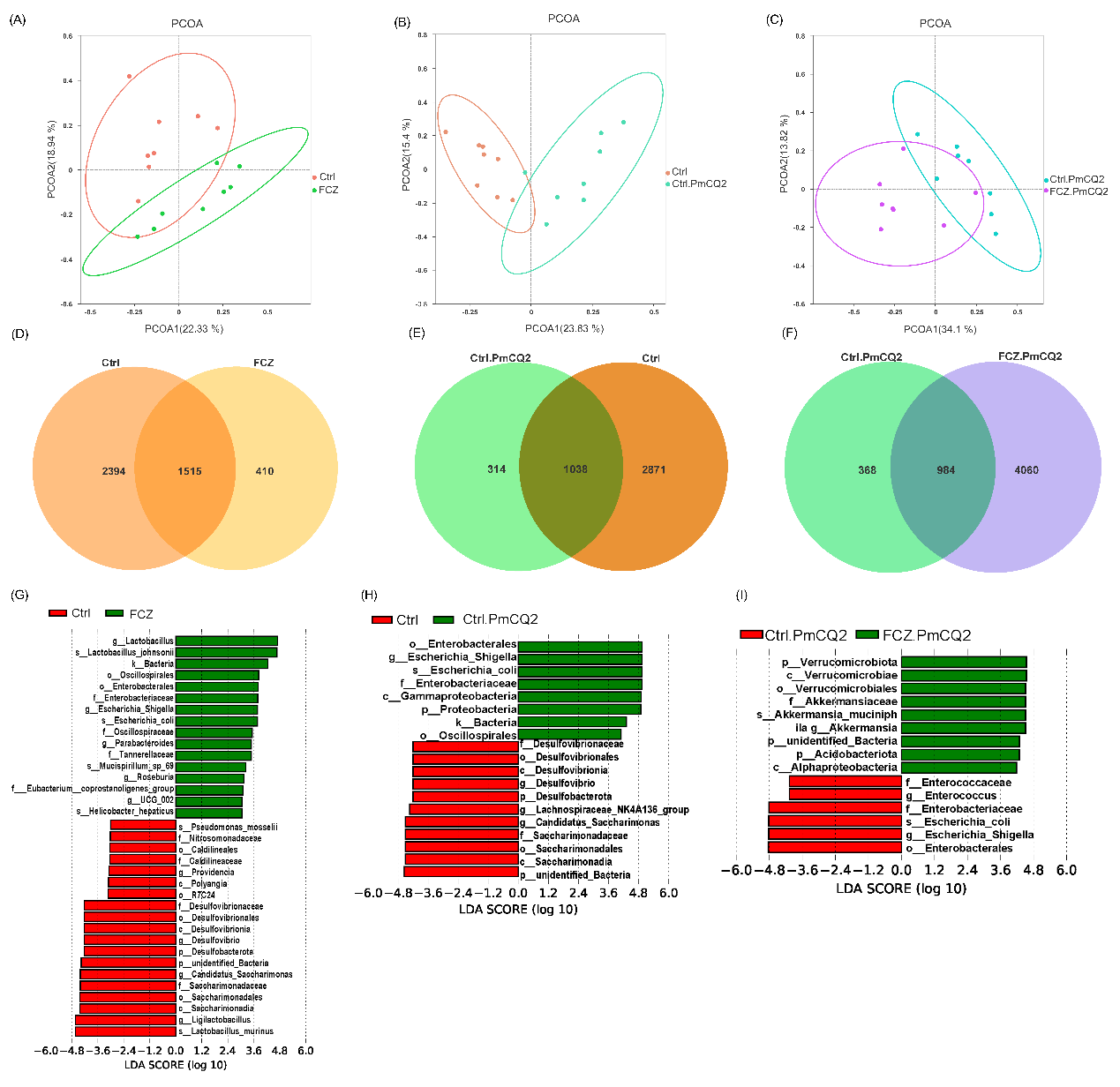


**Figure S4** Fluconazole treatment affects intestinal bacteria. (A-C) PCoA analysis of bacterial communities in indicated groups (*n* = 8). (D-F) Venn analysis of bacterial communities in indicated groups (*n* = 8). (G-I) LEfSe analysis on sample in indicated groups (LDA Score ≥ 4; *n* = 8).


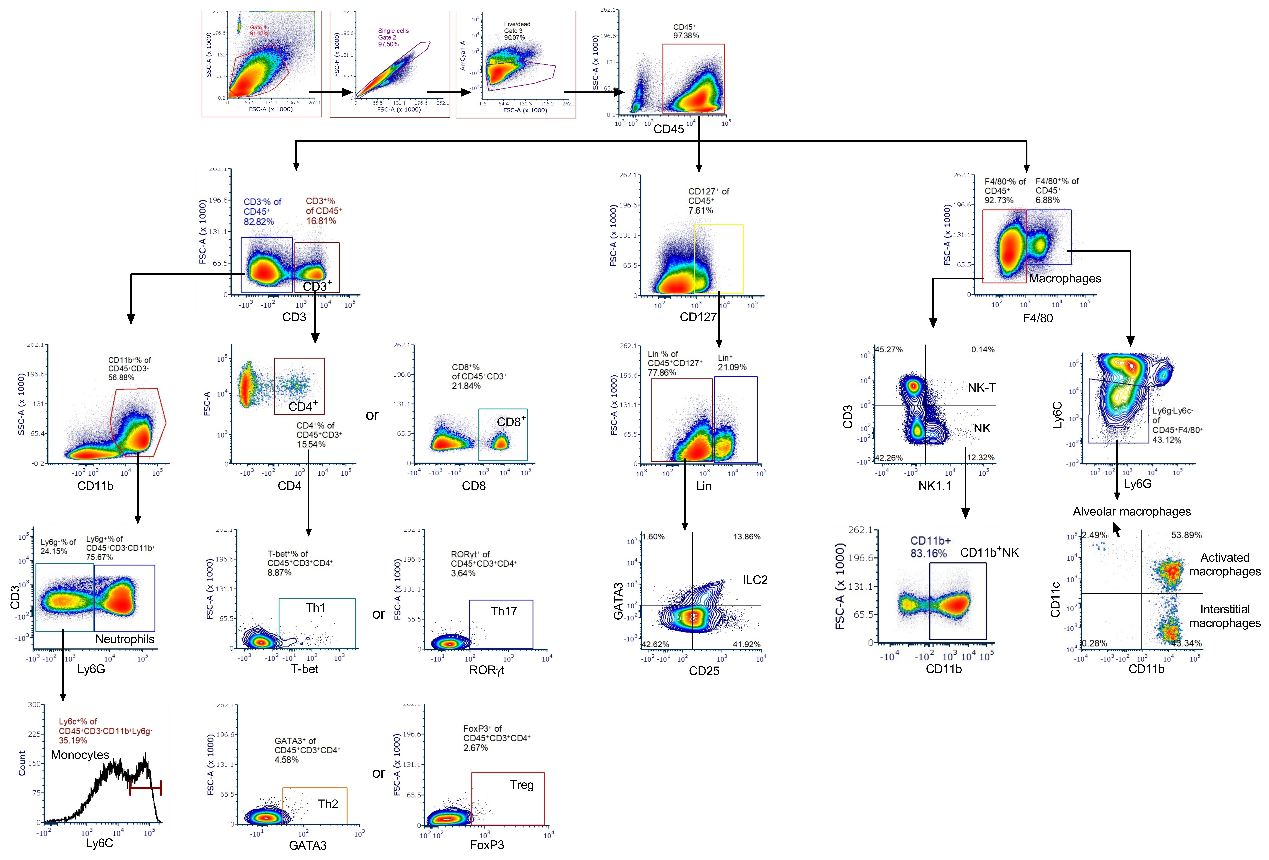


**Figure S5** Gate strategy of flow cytometry. Single cell suspensions from mouse lung tissue are analyzed with following gating strategies: Single cell gate, Live/dead gate, CD45 gate. And then, neutrophils were gated with CD3^-^CD11b^+^Ly6G^+^; monocytes were gated with CD3^-^CD11b^+^Ly6G^-^Ly6C^+^. T cells were gated with CD3^+^CD4^+^T-bet^+^ as Th1 group; CD3^+^CD4^+^RORγt^+^ as Th17 group; CD3^+^CD4^+^GATA3^+^ as Th2 group; CD3^+^CD4^+^FoxP3^+^ as Treg group; or CD3^+^CD8^+^ T cell group. ILC2 were gated with CD127^+^Lin^-^CD25^+^GATA3^+^. NK cells were gated with F4/80^-^, and NK1.1^+^CD3^+^ gated as NK-T cell; NK1.1^+^CD3^-^ gated as NK cell; NK1.1^+^CD3^-^CD11b^+^ gated as CD11b^+^NK cell. Macrophages were gated with F4/80^+^Ly6G^-^Ly6C^-^, and CD11b^-^CD11c^+^ gated as alveolar macrophages; CD11b^+^CD11c^+^ gated as activated macrophages; CD11b^+^CD11c^-^ gated as interstitial macrophages.


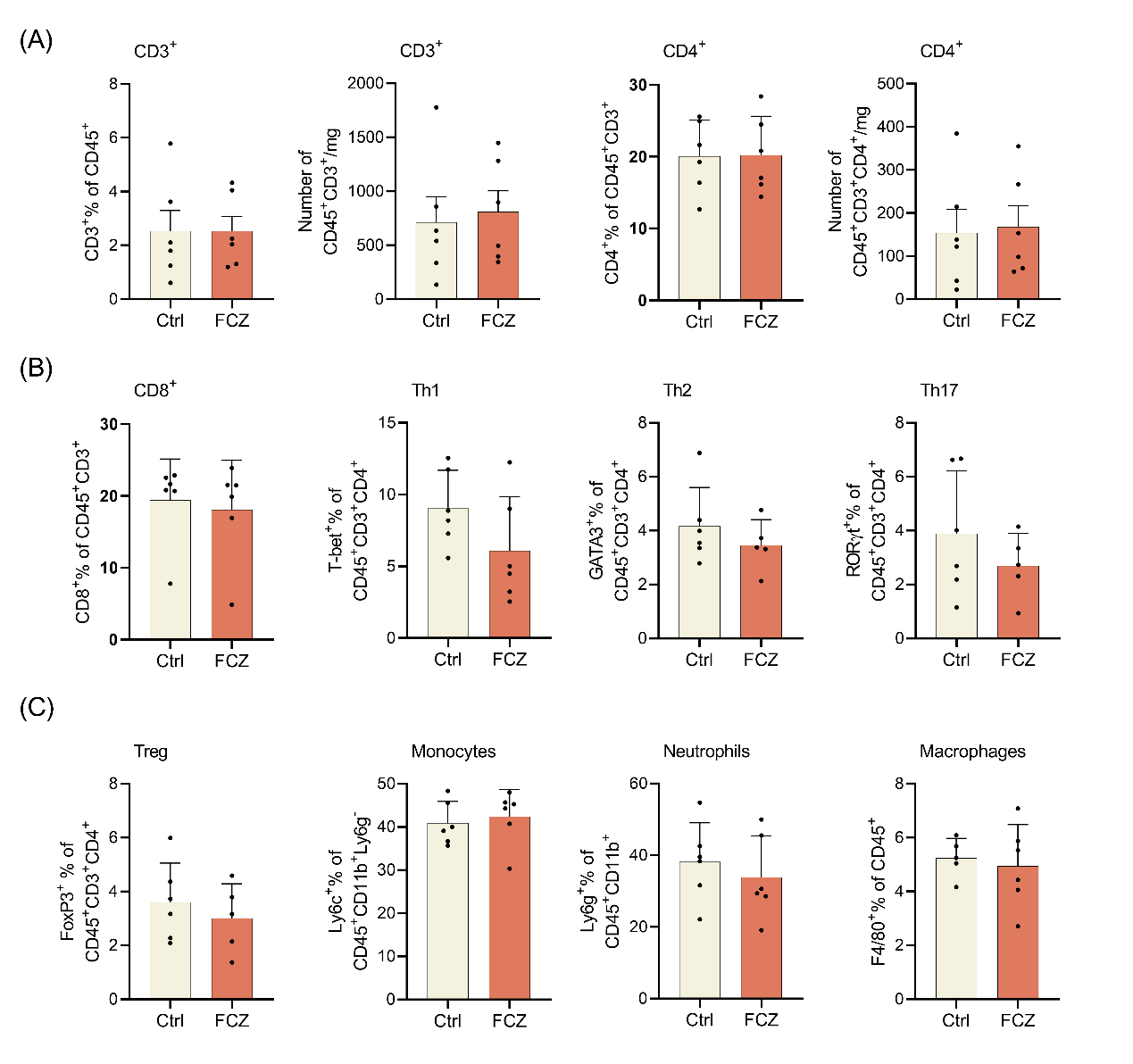


**Figure S6** Effect of intestinal mycobiota on lung immune cells. (A-C) Flow cytometry analysis of CD3^+^, CD4^+^, CD8^+^, Th1 (T-bet^+^), Th2 (GATA3^+^), Th17 (RORγt^+^), Treg (FoxP3^+^) cells, monocytes (Ly6C^+^), neutrophils (Ly6G^+^), and macrophages (F4/80^+^) in the lung. CD3^+^ cells were pregated by CD45^+^; CD4^+^ and CD8^+^cells were pregated by CD45^+^CD3^+^; Th1 (T-bet^+^), Th2 (GATA3^+^), Th17 (RORγt^+^), Treg (FoxP3^+^) cells were pregated by CD45^+^CD3^+^CD4^+^, nonocytes were pregated by CD45^+^CD11b^+^Ly6G^-^ and neutrophils were pregated by CD45^+^CD11b^+^ (*n* = 5 and 6). Data were analyzed by unpaired *t* test and represented as mean ± SD unless indicated. **p* < 0.05, ***p* < 0.01, ****p* < 0.001, and *****p* < 0.0001.

**
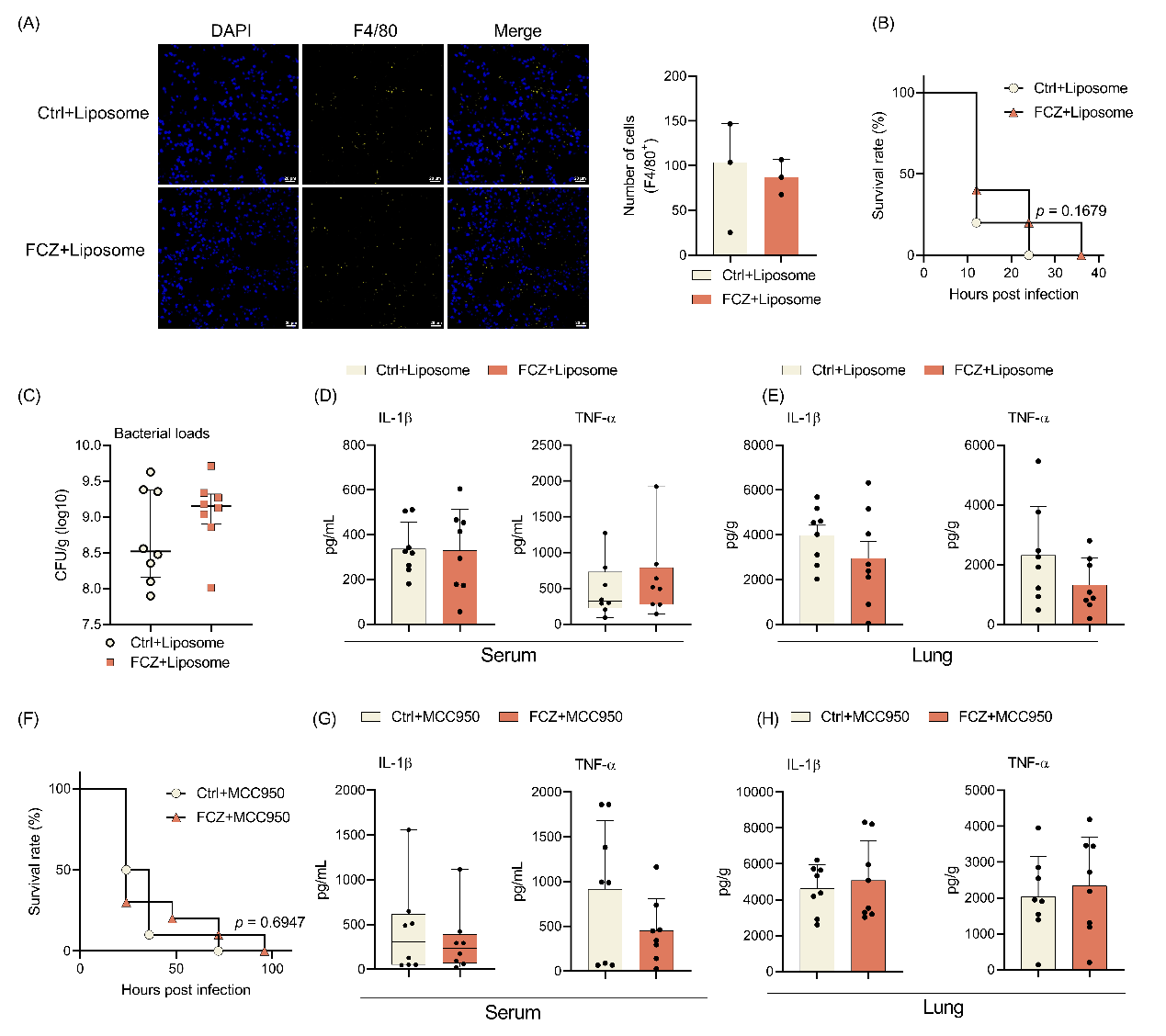
**

**Figure S7** Deletion of macrophages relieves lung inflammation during infection. (A) Immunofluorescence analysis of F4/80^+^ cells (*n* = 3, Scale bar, 20 μm). The number of F4/80^+^ cells was analyzed by Mann-Whitney *U* test with data shown as M(IQR). (B) The survival rate of mice (*n* = 10). (C) Bacterial burdens of mouse lung analyzed by Mann-Whitney *U* test. Data were shown as M(IQR) (*n* = 8). (D-E) The levels of IL-1β and TNF-α in the serum and lung with data of serum TNF-α analyzed by Mann-Whitney *U* test and shown as M(IQR) (*n* = 8). (F) The survival rate of mice (*n* = 10). (G-H) The levels of IL-1β and TNF-α in the serum and lung with data of serum IL-1β analyzed by Mann-Whitney *U* test and shown as M(IQR) (*n* = 8). Data were analyzed by unpaired *t* test and represented as mean ± SD unless indicated. **p* < 0.05, ***p* < 0.01, ****p* < 0.001, and *****p* < 0.0001.


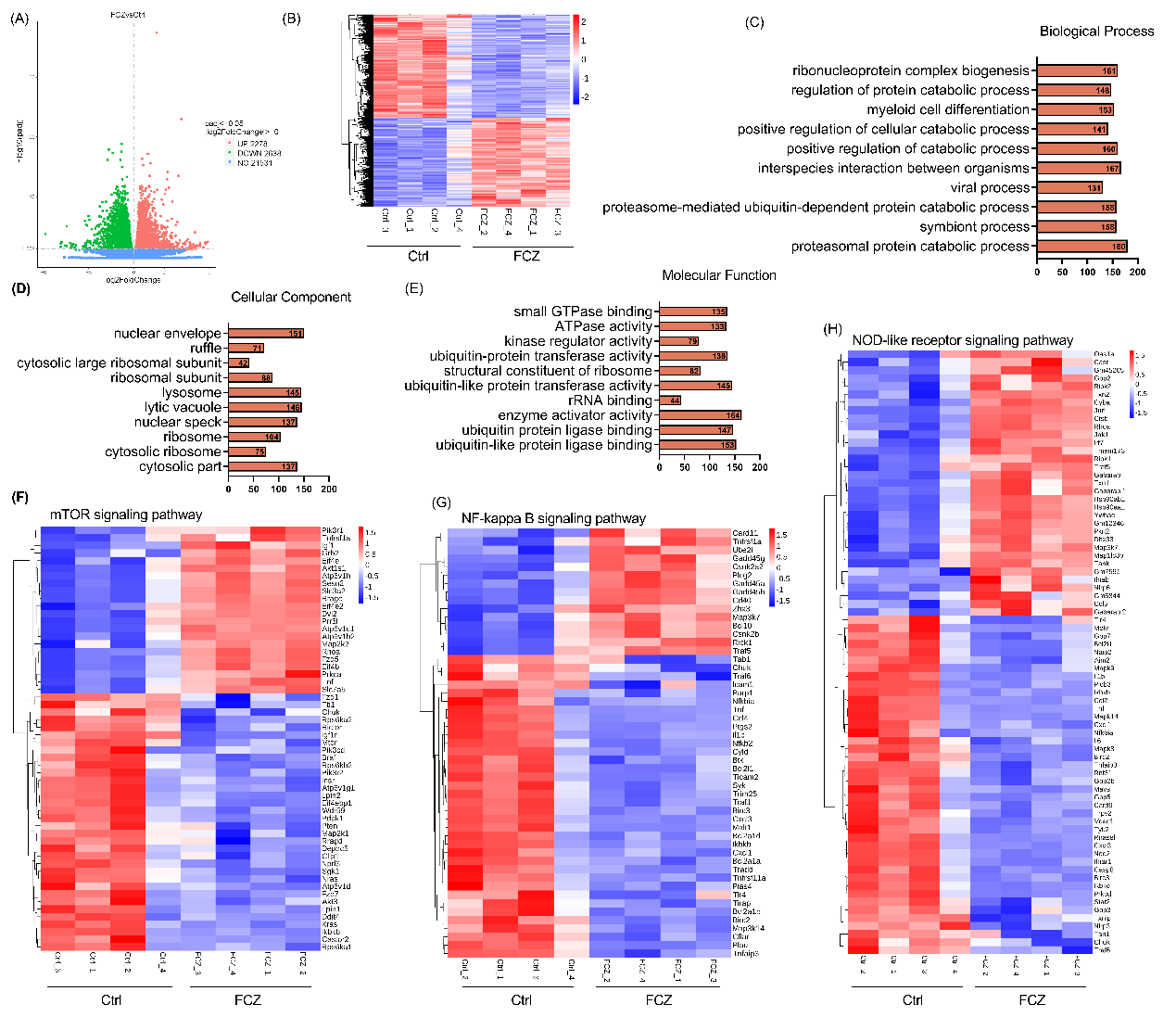


**Figure S8** *E. coli* activates the immune response of macrophages. (A) Volcano plot analysis of all genes in PEMs (*n* = 4). (B) Heatmap analysis of up-regulated (red) or down-regulated genes (blue) in PEMs (*n* = 4). (C-E) Functional enrichment analysis of top 10 pathway in biological process (C), cellular component (D) and molecular function (E) (*n* = 4). (F-H) Heatmap analysis of the DEGs in “mTOR signaling pathway”, “NF-kappa B signaling pathway”, and “NOD-like receptor signaling pathway” (*n* = 4). *padj* < 0.05.


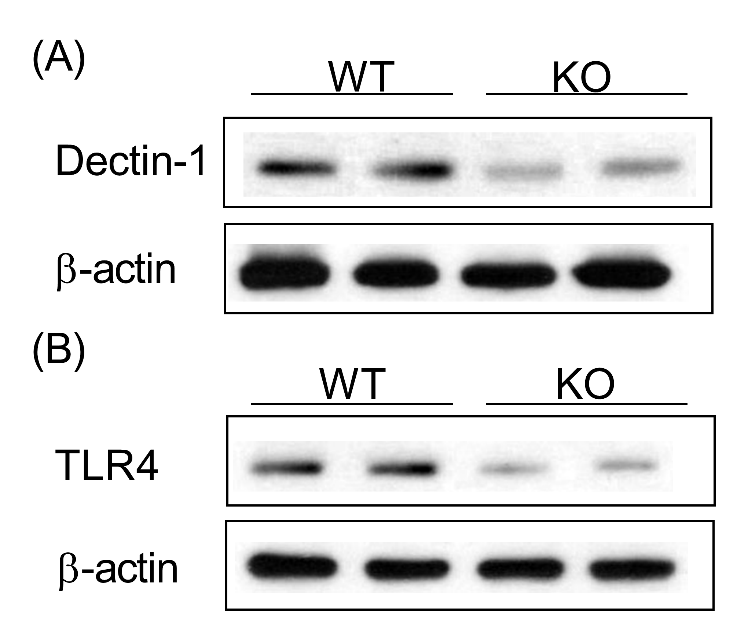


**Figure S9** Western blots analysis of Dectin-1 and TLR4. (A) Protein expression of Dectin-1 (*n* = 2). (B) Protein expression of TLR4 (*n* = 2).

**Source data**

**Immunofluorescence**

| Figure 3M | 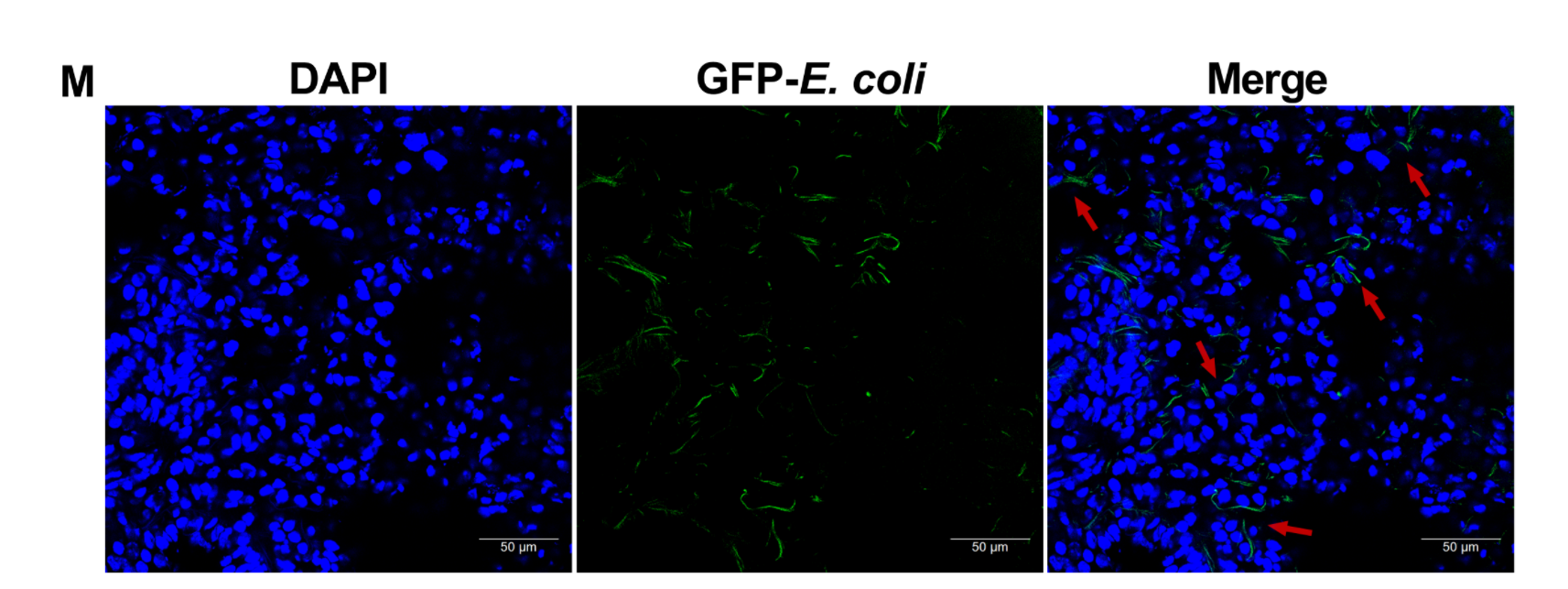 | | |
| --- | --- | --- | --- |
|  | **Merge** | **DAPI** | **GFP-labeled *E. coli*** |
| 1 | 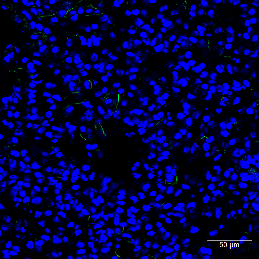 | 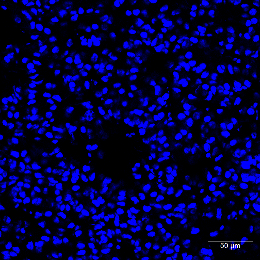 | 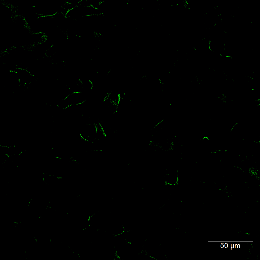 |
| 2 | 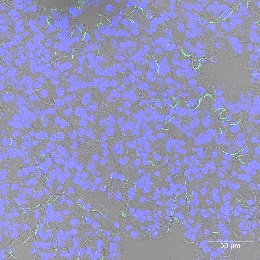 | 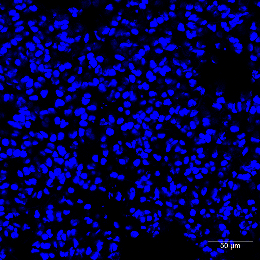 | 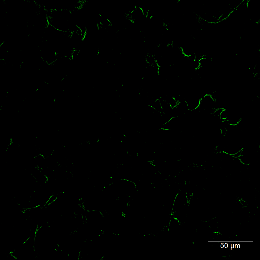 |
| 3 | 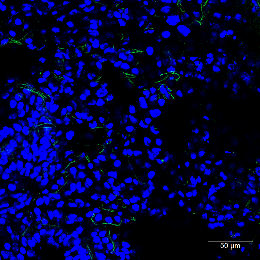 | 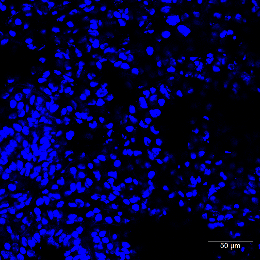 | 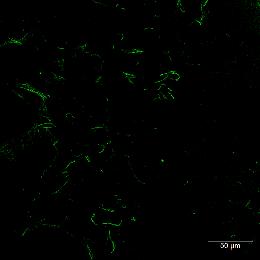 |
| 4 | 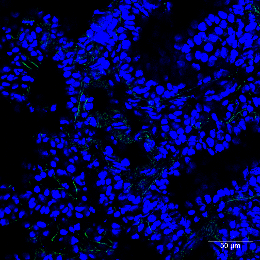 | 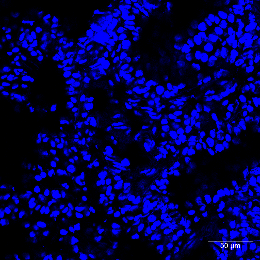 | 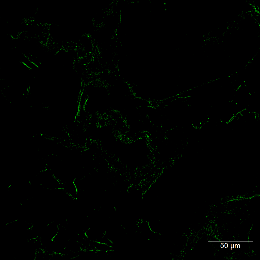 |
| 5 | 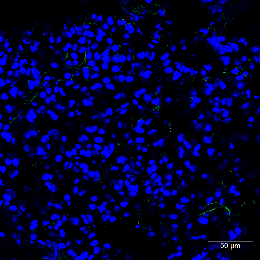 | 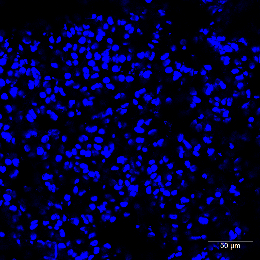 | 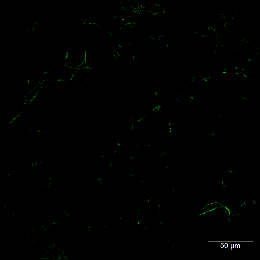 |
| 6 | 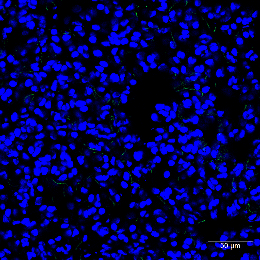 | 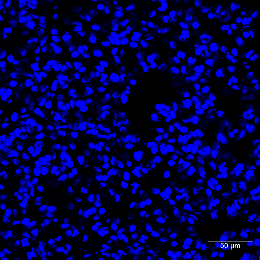 | 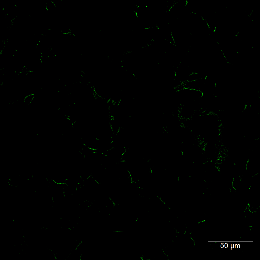 |

| **Figure 4 G** | **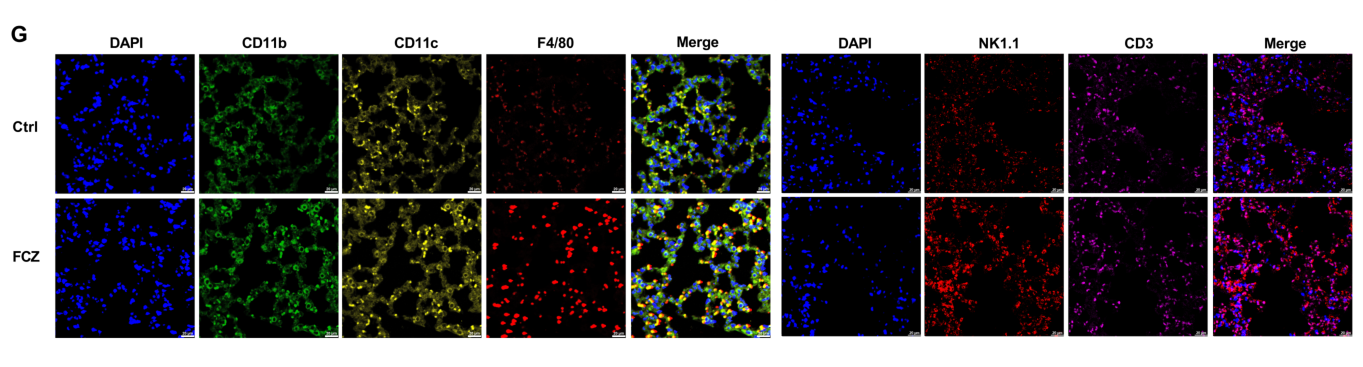** | | | | |
| --- | --- | --- | --- | --- | --- |
|  | **Merge** | **DAPI** | **CD11b** | **CD11c** | **F4/80** |
| Ctrl 1-1 | 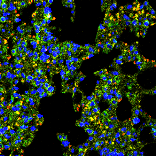 | 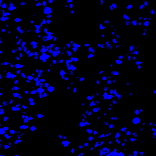 | 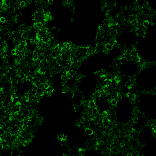 | 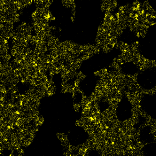 | 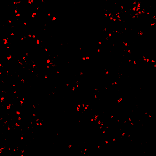 |
| Ctrl 1-2 | 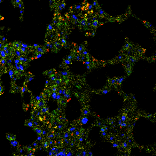 | 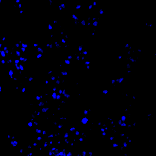 | 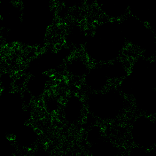 | 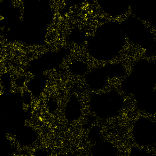 | 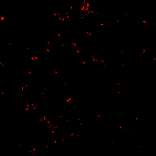 |
| Ctrl 1-3 | 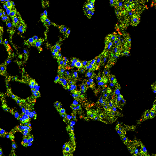 | 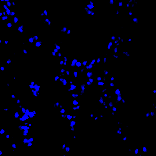 | 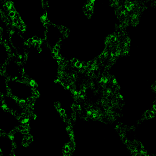 | 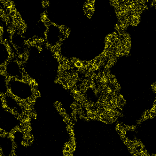 | 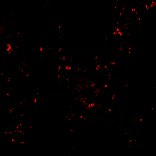 |
| Ctrl 1-4 | 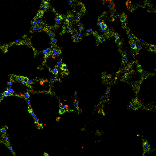 | 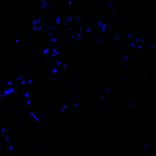 | 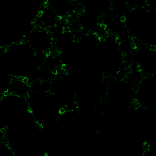 | 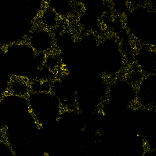 | 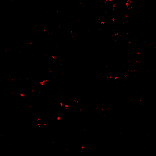 |
| Ctrl 1-5 | 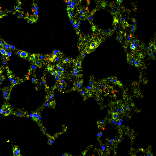 | 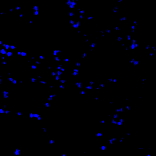 | 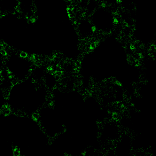 | 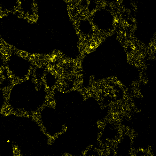 | 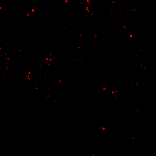 |
| Ctrl 1-6 | 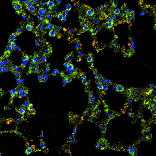 | 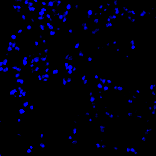 | 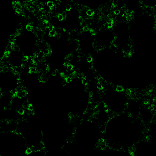 | 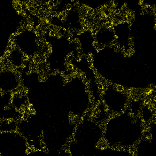 | 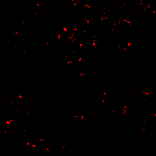 |
| Ctrl 1-7 | 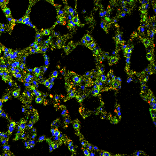 | 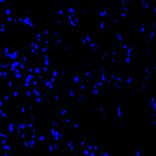 | 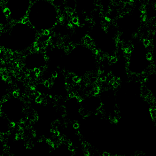 | 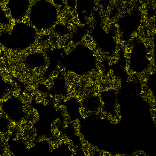 | 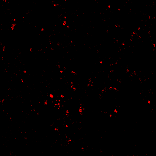 |
| Ctrl 2-1 | 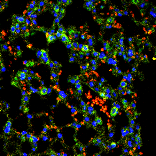 | 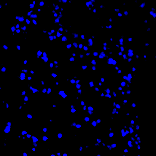 | 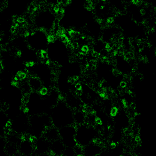 | 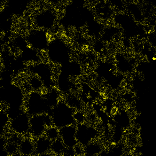 | 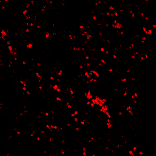 |
| Ctrl 2-2 | 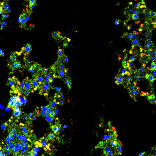 | 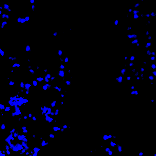 | 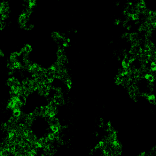 | 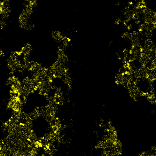 | 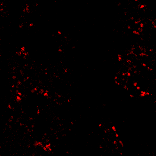 |
| Ctrl 2-3 | 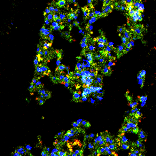 | 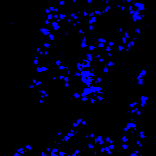 | 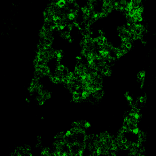 | 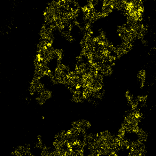 | 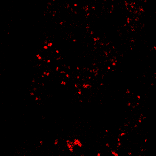 |
| Ctrl 2-4 | 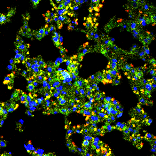 | 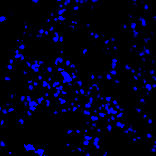 | 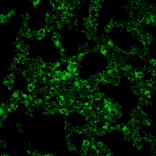 | 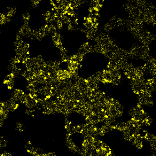 | 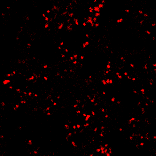 |
| Ctrl 2-5 | 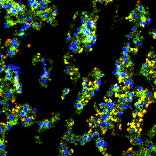 | 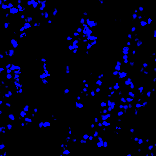 | 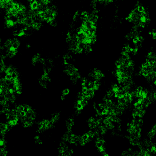 | 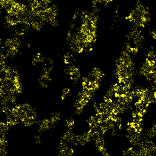 | 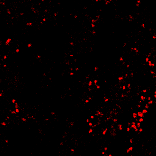 |
| Ctrl 2-6 | 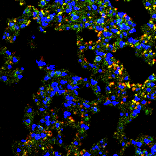 | 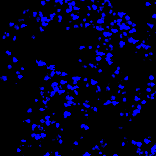 | 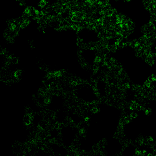 | 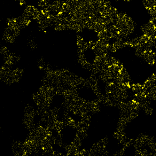 | 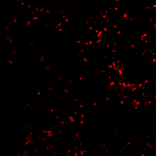 |
| Ctrl 3-1 | 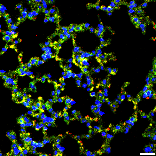 | 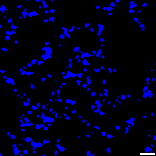 | 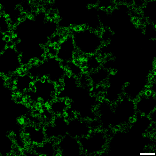 | 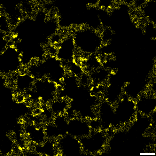 | 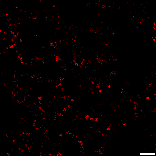 |
| Ctrl 3-2 | 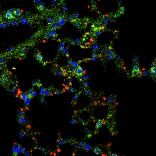 |  |  |  |  |
| Ctrl 3-3 |  |  |  |  |  |
| Ctrl 3-4 |  |  |  |  |  |
| Ctrl 3-5 |  |  |  |  |  |
| Ctrl 3-6 |  |  |  |  |  |
| FCZ 1-1 |  |  |  |  |  |
| FCZ 1-2 |  |  |  |  |  |
| FCZ 1-3 |  |  |  |  |  |
| FCZ 1-4 |  |  |  |  |  |
| FCZ 1-5 |  |  |  |  |  |
| FCZ 1-6 |  |  |  |  |  |
| FCZ 2-1 |  |  |  |  |  |
| FCZ 2-2 |  |  |  |  |  |
| FCZ 2-3 |  |  |  |  |  |
| FCZ 2-4 |  |  |  |  |  |
| FCZ 2-5 |  |  |  |  |  |
| FCZ 2-6 |  |  |  |  |  |
| FCZ 2-7 |  |  |  |  |  |
| FCZ 3-1 |  |  |  |  |  |
| FCZ 3-2 |  |  |  |  |  |
| FCZ 3-3 |  |  |  |  |  |
| FCZ 3-4 |  |  |  |  |  |
| FCZ 3-5 |  |  |  |  |  |
| FCZ 3**-6** |  |  |  |  |  |
| FCZ 3**-7** |  |  |  |  |  |
|  |  |  |  |  |  |

| **Figure 4 G** |  | | | |
| --- | --- | --- | --- | --- |
|  | **Merge** | **DAPI** | **CD3** | **NK1.1** |
| Ctrl 1-1 |  |  |  |  |
| Ctrl 1-2 |  |  |  |  |
| Ctrl 1-3 |  |  |  |  |
| Ctrl 1-4 |  |  |  |  |
| Ctrl 1-5 |  |  |  |  |
| Ctrl 1-6 |  |  |  |  |
| Ctrl 1-7 |  |  |  |  |
| Ctrl 1-8 |  |  |  |  |
| Ctrl 1-9 |  |  |  |  |
| Ctrl 2-1 |  |  |  |  |
| Ctrl 2-2 |  |  |  |  |
| Ctrl 2-3 |  |  |  |  |
| Ctrl 2-4 |  |  |  |  |
| Ctrl 2-5 |  |  |  |  |
| Ctrl 3-1 |  |  |  |  |
| Ctrl 3-2 |  |  |  |  |
| Ctrl 3-3 |  |  |  |  |
| Ctrl 3-4 |  |  |  |  |
| Ctrl 3-5 |  |  |  |  |
| FCZ 1-1 |  |  |  |  |
| FCZ 1-2 |  |  |  |  |
| FCZ 1-3 |  |  |  |  |
| FCZ 1-4 |  |  |  |  |
| FCZ 2-1 |  |  |  |  |
| FCZ 2-2 |  |  |  |  |
| FCZ 2-3 |  |  |  |  |
| FCZ 2-4 |  |  |  |  |
| FCZ 2-5 |  |  |  |  |
| FCZ 2-6 |  |  |  |  |
| FCZ 3-1 |  |  |  |  |
| FCZ 3-2 |  |  |  |  |
| FCZ 3-3 |  |  |  |  |
| FCZ 3-4 |  |  |  |  |
| FCZ 3-5 |  |  |  |  |
| FCZ 3-6 |  |  |  |  |
| FCZ 3-7 |  |  |  |  |
|  |  |  |  |  |

| Figure S7A |  | | |
| --- | --- | --- | --- |
|  | **Merge** | **DAPI** | **F4/80** |
| Ctrl+Liposome 1-1 |  |  |  |
| Ctrl+Liposome 1-2 |  |  |  |
| Ctrl+Liposome 1-3 |  |  |  |
| Ctrl+Liposome 1-4 |  |  |  |
| Ctrl+Liposome 1-5 |  |  |  |
| Ctrl+Liposome 1-6 |  |  |  |
| Ctrl+Liposome 2-1 |  |  |  |
| Ctrl+Liposome 2-2 |  |  |  |
| Ctrl+Liposome 2-3 |  |  |  |
| Ctrl+Liposome 2-4 |  |  |  |
| Ctrl+Liposome 2-5 |  |  |  |
| Ctrl+Liposome 24-6 |  |  |  |
| Ctrl+Liposome 2-7 |  |  |  |
| Ctrl+Liposome 2-8 |  |  |  |
| Ctrl+Liposome 2-9 |  |  |  |
| Ctrl+Liposome 3-1 |  |  |  |
| Ctrl+Liposome 3-2 |  |  |  |
| Ctrl+Liposome 3-3 |  |  |  |
| Ctrl+Liposome 3-4 |  |  |  |
| Ctrl+Liposome 3-5 |  |  |  |
| Ctrl+Liposome 3-6 |  |  |  |
| Ctrl+Liposome 3-7 |  |  |  |
| Ctrl+Liposome 3-8 |  |  |  |
| Ctrl+Liposome 3-9 |  |  |  |
| FCZ+Liposome 1-1 |  |  |  |
| FCZ+Liposome 1-2 |  |  |  |
| FCZ+Liposome 1-3 |  |  |  |
| FCZ+Liposome 1-4 |  |  |  |
| FCZ+Liposome 1-5 |  |  |  |
| FCZ+Liposome 1-6 |  |  |  |
| FCZ+Liposome 1-7 |  |  |  |
| FCZ+Liposome 2-1 |  |  |  |
| FCZ+Liposome 2-2 |  |  |  |
| FCZ+Liposome 2-3 |  |  |  |
| FCZ+Liposome 2-4 |  |  |  |
| FCZ+Liposome 2-5 |  |  |  |
| FCZ+Liposome 2-6 |  |  |  |
| FCZ+Liposome 2-7 |  |  |  |
| FCZ+Liposome 3-1 |  |  |  |
| FCZ+Liposome 3-2 |  |  |  |
| FCZ+Liposome 3-3 |  |  |  |
| FCZ+Liposome 3-4 |  |  |  |
| FCZ+Liposome 3-5 |  |  |  |
| FCZ+Liposome 3-6 |  |  |  |

| Figure 5D |  | | | |
| --- | --- | --- | --- | --- |
|  | **Merge** | **DAPI** | **NLRP3** | **ASC** |
| Ctrl 1-1 |  |  |  |  |
| Ctrl 1-2 |  |  |  |  |
| Ctrl 1-3 |  |  |  |  |
| Ctrl 1-4 |  |  |  |  |
| Ctrl 2-1 |  |  |  |  |
| Ctrl 2-2 |  |  |  |  |
| Ctrl 2-3 |  |  |  |  |
| Ctrl 2-4 |  |  |  |  |
| Ctrl 3-1 |  |  |  |  |
| Ctrl 3-2 |  |  |  |  |
| Ctrl 3-3 |  |  |  |  |
| FCZ 1-1 |  |  |  |  |
| FCZ 1-2 |  |  |  |  |
| FCZ 1-3 |  |  |  |  |
| FCZ 2-1 |  |  |  |  |
| FCZ 2-2 |  |  |  |  |
| FCZ 2-3 |  |  |  |  |
| FCZ 3-1 |  |  |  |  |
| FCZ 3-2 |  |  |  |  |
|  | **Merge** | **DAPI** | **Caspase-1** |  |
| Ctrl 1-1 |  |  |  |  |
| Ctrl 1-2 |  |  |  |  |
| Ctrl 1-3 |  |  |  |  |
| Ctrl 2-1 |  |  |  |  |
| Ctrl 2-2 |  |  |  |  |
| Ctrl 2-3 |  |  |  |  |
| Ctrl 3-1 |  |  |  |  |
| Ctrl 3-2 |  |  |  |  |
| Ctrl 3-3 |  |  |  |  |
| FCZ 1-1 |  |  |  |  |
| FCZ 1-2 |  |  |  |  |
| FCZ 2-1 |  |  |  |  |
| FCZ 2-2 |  |  |  |  |
| FCZ 2-3 |  |  |  |  |
| FCZ 3-1 |  |  |  |  |
| FCZ 3-2 |  |  |  |  |
| FCZ 3-3 |  |  |  |  |

**Western blot**

| Figure 5E |  |
| --- | --- |
| **NLRP3 (110 KDa)** |  |
| **ASC (22 KDa)** |  |
| **Caspase-1 (45 KDa)** |  |
| **Caspase-1 (42 KDa)** |  |
| **Caspase-1 (35 KDa)** |  |
| **Caspase-1 (12 KDa)** |  |
| **Caspase-1 (10 KDa)** |  |
| **p65** |  |
| **p-p65** |  |
| **IL-1β (31 KDa)** |  |
| **IL-1β (17 KDa)** |  |
| **β-actin** |  |
|  |  |

| Figure S9 |  |
| --- | --- |
| **Dectin-1** |  |
| **β-actin** |  |
| **TLR-4** |  |
| **β-actin** |  |
|  |  |
